# Epigenomic footprints across 111 reference epigenomes reveal tissue-specific epigenetic regulation of lincRNAs

**DOI:** 10.1038/ncomms7370

**Published:** 2015-02-18

**Authors:** Viren Amin, R. Alan Harris, Vitor Onuchic, Andrew R. Jackson, Tim Charnecki, Sameer Paithankar, Sai Lakshmi Subramanian, Kevin Riehle, Cristian Coarfa, Aleksandar Milosavljevic

**Affiliations:** 1 Epigenome Center, Bioinformatics Research Laboratory, Department of Molecular & Human, Genetics, Baylor College of Medicine, One Baylor Plaza, BCMD 400D, Houston, Texas 77030, USA

## Abstract

Tissue-specific expression of lincRNAs suggests developmental and cell-type-specific functions, yet tissue specificity was established for only a small fraction of lincRNAs. Here, by analysing 111 reference epigenomes from the NIH Roadmap Epigenomics project, we determine tissue-specific epigenetic regulation for 3,753 (69% examined) lincRNAs, with 54% active in one of the 14 cell/tissue clusters and an additional 15% in two or three clusters. A larger fraction of lincRNA TSSs is marked in a tissue-specific manner by H3K4me1 than by H3K4me3. The tissue-specific lincRNAs are strongly linked to tissue-specific pathways and undergo distinct chromatin state transitions during cellular differentiation. Polycomb-regulated lincRNAs reside in the bivalent state in embryonic stem cells and many of them undergo H3K27me3-mediated silencing at early stages of differentiation. The exquisitely tissue-specific epigenetic regulation of lincRNAs and the assignment of a majority of them to specific tissue types will inform future studies of this newly discovered class of genes.

## Abstract (web summary)

Tissue-specific functions have been established for some lincRNAs. Here, by analysing 111 reference epigenomes from the NIH Roadmap Epigenomics project, the authors report tissue-specific epigenomic regulation of 3,753 lincRNAs and their strong connection with tissue-specific pathways.

## Graphical abstract

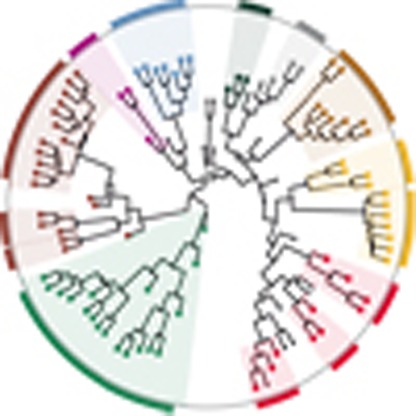

Long noncoding RNAs (lncRNAs) are implicated in an increasing number of cellular processes including mammalian cellular differentiation^1^. Their role in repressing lineage-specific genes during early development was demonstrated by knockdown experiments in mouse embryonic stem cells (ESCs)^2^. Lineage-specific role of specific lncRNAs has now been established in cardiac^3^,^4^, epidermal^5^, neuronal^6^, mammary gland development^7^,^8^ and in T cells^9^. Striking tissue-specific transcription of lncRNAs^10^,^11^ is consistent with their role in developmental regulation and presents a possible inroad into understanding their biology.

The intergenic lncRNAs (lincRNAs) are a major class of lncRNAs that are particularly convenient to study computationally and experimentally because of their lack of overlap with protein-coding genes. Despite their relative accessibility, lincRNAs are experimentally less tractable than protein-coding genes because of the lack of information about their potential function and associated phenotypes. We here address this knowledge gap by determining their tissue-specific epigenetic regulation, thus complementing the current knowledge about their tissue-specific transcription.

Since lincRNA transcript levels are much lower than those for protein-coding genes, precise characterization of lincRNAs requires either highly sensitive assays of transcription initiation^12^ or very deep RNA sequencing^11^. Epigenomic footprints provide additional insights into lincRNA regulation. A major impetus in lincRNA research came through the discovery that a combination of a punctate H3K4me3 mark over lincRNA transcription start site (TSS) and a flanking broad segments marked by H3K36me3—an epigenomic footprint of active transcription—is informative for identifying not only actively transcribed protein-coding genes but also active lincRNAs^11^,^13^. While some reports suggest that histone modifications associated with lincRNA genes cannot be distinguished from those associated with protein-coding genes^14^, others suggest that a large fraction of lincRNAs associates with H3K4me1^15^, a histone mark previously associated with active enhancers^16^. We here address this open question by performing a comprehensive analysis of epigenomic footprints at lincRNA promoters across a large diversity of cell types.

The role of lincRNAs in cellular differentiation is underscored by their multiple associations with Polycomb group (PcG) proteins. Epigenetic PcG-mediated repression of lncRNAs is suggested by experiments that show derepression of lncRNAs in ESCs on knockdown of the PRC2 member *Ezh2* (ref. 17). The lncRNAs themselves facilitate H3K27me3-mediated Polycomb regulation^18^. PcG proteins interact with several well-studied mammalian lncRNAs, including X silencing regulator *Xist*, imprinting regulator *Kcnq1ot1* and transcriptional regulator *HOTAIR*. Recent studies indicate that a large fraction (~20%) of lncRNAs physically associates with the members of the PRC2 complex^19^. Despite these initial results, the role of lincRNAs in Polycomb-mediated regulation of cellular differentiation and in particular their relation to developmentally central bivalent states^20^ remains poorly understood.

Overall, current reports indicate strong links between epigenetic regulation and lincRNA, but the reported findings remain fragmentary and sometimes contradictory. Moreover, because of limited diversity of cell and tissue types profiled, for a vast majority of lincRNAs we still do not know the specific tissue or cell type where the lincRNA is likely to be active. To address these open questions, we here examine epigenomic footprints of lincRNAs across 111 reference epigenomes within Release 9 of the Human Epigenome Atlas, a product of the NIH Roadmap Epigenomics project^21^. The reference epigenomes represent 99 distinct human cell and tissue types, by far the most diverse collection examined for this purpose so far and is the first ever to use chromatin states to assess tissue specificity of lincRNAs. Each of the reference epigenomes reported here contains profiles of five core histone marks (H3K4me3, H3K4me1, H3K27me3, H3K9me3 and H3K36me3), thus enabling unbiased and comprehensive examination of cell- and tissue-specific epigenomic footprints of lincRNAs and their comparison with epigenomic footprints of enhancers, promoters and other regulatory elements.

In the following, we pursue a three-stage analysis. First, we identify epigenomic features that discriminate established cell- and tissue-types. We find that dynamic epigenomic footprints at lincRNA TSSs are at least as tissue specific as the footprints of enhancers and significantly more specific than those of promoters. Second, using their epigenomic footprints, we assign a fraction of promoters, a majority of enhancers and even larger fraction of lincRNA TSSs to specific tissue types and discover striking association of those tissue-specific regulatory elements with cell- and tissue-specific developmental processes and mammalian phenotypes. Finally, we examine patterns of epigenetic programming of regulatory elements as cells differentiate, with a focus on Polycomb regulation of lincRNAs.

## Results

### Epigenomic footprints discriminate cell types

As regulatory elements acquire cell- and tissue-specific histone marks on cellular differentiation, we reasoned that histone marks over regulatory elements may provide sufficient information to discriminate major cell- and tissue types. To test this hypothesis, we performed clustering of 99 distinct cell types represented within 111 reference epigenomes from the Release 9 of the Human Epigenome Atlas (see Methods). The clustering was performed using average signals of five core histone marks over eight types of regulatory elements, including enhancers, promoters and lincRNA TSSs (Supplementary Fig. 1).

**Figure 1:**
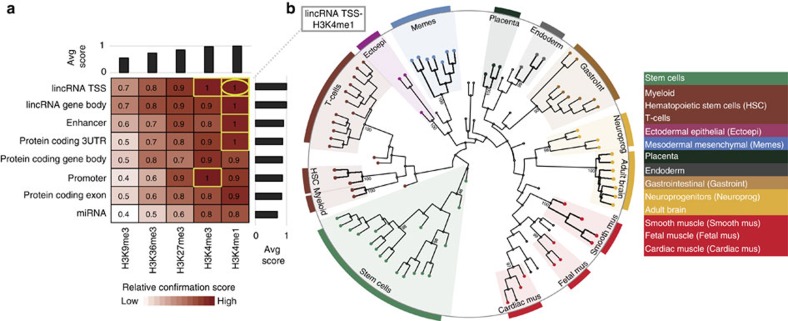
Clustering of epigenomes. (**a**) Hierarchical clustering of 99 cell and tissue types was performed using 40 different combinations of five histone modifications (columns) and eight groups of regions of interests (rows). A high score for a particular combination (histone mark—region of interest score—number rounded to a single decimal place) indicates that the subtrees of a tree constructed using the combination are frequently confirmed across 40 combinations. The combinations with highest scores are deemed most informative and are highlighted in yellow. Column bars and row bars indicate average informativeness of specific histone modifications and regions of interests, respectively. (**b**) Circular dendrogram constructed using the top-scoring combination indicated by yellow circle in panel **a** (average H3K4me1 histone modification signal over 3,000 bp windows centred on lincRNA transcription start sites). The highlighted sections in the dendrogram correspond to fourteen major clusters and nine groups of clusters (indicated by nine distinct colours) corresponding to related cell- and tissue-types. The coloured sections correspond to clusters that are highly reproducible (high bootstrap scores, see Supplementary Fig. 2) and can be derived using different combinations of histone marks and regions of interest.

We reasoned that the clusters of samples that share epigenomic features and cellular identity acquired during differentiation would emerge repeatedly from trees generated by independent mark-ROI (histone mark-region of interests) combinations. To identify such sample clusters, a confirmation score was defined for any particular cluster as the fraction of trees that have a subtree corresponding to that sample cluster (for details, see Methods). To identify the trees that most closely track cellular identity, confirmation score was computed for whole trees by averaging scores for all of its subtrees. As illustrated in Fig. 1a, mark-ROI combinations that are known to be highly informative for determining cell-type identity, such as H3K4me1 at enhancers and H3K4me3 at promoters, indeed stood out with highest confirmation scores.

The trees produced using lincRNA TSSs achieved overall highest confirmation scores among all the ROIs tested, indicating the highest degree of tissue-specific epigenetic regulation. High confirmation scores were achieved for lincRNA TSSs both in combination with H3K4me3, a signature mark of active promoters and with H3K4me1, a mark commonly associated with active enhancers (Fig. 1a), suggesting importance of both marks in tissue-specific epigenetic regulation of lincRNAs.

The tree obtained using the H3K4me1 mark over lincRNA TSSs is represented in Fig. 1b. Highlighted in the figure are the fourteen clusters that closely correspond to known cell- and tissue types. These clusters show high reproducibility, as indicated by both high confirmation scores and high bootstrap scores (Supplementary Figs 2 and 3 and Supplementary Table 1). We therefore conclude that histone marks at regulatory elements, and particularly the H3K4me1 mark at lincRNA TSSs, provide information sufficient to discriminate major cell and tissue types and indicate high degree of epigenetic regulation of cellular identity, particularly at lincRNA TSSs.

### Cell-type-specific epigenomic footprints of lincRNAs

**Figure 2:**
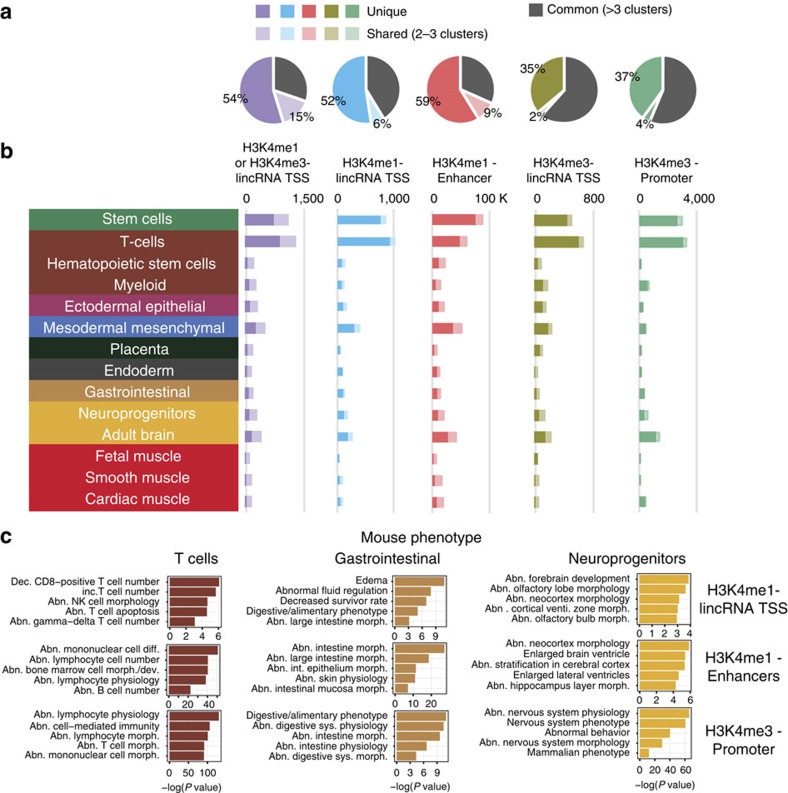
Lineage-specific regulatory regions and associated phenotypes. (**a**) Lineage-specific regulatory regions were determined by comparing epigenomes within a cluster against the epigenomes outside the cluster using linear regression fit modelling (LIMMA^22^, *P* value<0.05). Pie chart shows percentage of regions that harbour cluster-specific marks, some unique to the cluster, some shared by two or three related subtrees, and highlights regulatory regions that are less specifically modified (grey). (**b**) Distribution of regulatory regions that are unique and shared for each cluster. (**c**) Enrichment of mouse phenotype terms associated with lineage specific regulators calculated using a GREAT tool’s binomial approach (lineages representing all three germ layers were selected).

We next examined histone marks discriminating the 14 highly reproducible clusters highlighted in Fig. 1b. To identify the lincRNA loci and other regulatory regions that show cluster-specific histone mark signals, histone mark levels over the regions were compared for the samples within a cluster and those outside, using linear regression fit modelling (LIMMA^22^, *P* value<0.05; for more details see Methods). Accuracy of 5′ ends of the GENCODE defined lincRNAs were confirmed with CAGE tags^23^ and DNaseI hypersensitivity peaks being enriched at lincRNA TSS (Supplementary Fig. 4). As indicated in Fig. 2a,b, of all regulatory elements, lincRNA TSSs show most tissue-specific activity signatures and promoters of protein-coding genes show less tissue specificity than enhancers, consistent with previous findings^10^,^11^ (Supplementary Data set 1–4).

Considering all cluster-specific histone mark levels over lincRNA TSSs, we observe that H3K4me1 signals are more frequently lineage specific than H3K4me3 and a significant fraction of sites show cluster-specific signals for both marks (Supplementary Fig. 5d). Most lincRNA TSSs (69%) showed cluster-specific changes, including 54% showing change in a single cluster and a further 15% that were slightly less specific because they showed changes in two or three closely related clusters (Supplementary Table 2 and Supplementary Fig. 5a–c). There are also more cluster-specific chromatin mark signals in the gene bodies of lincRNAs than protein-coding genes (Supplementary Table 3). As most lncRNA TSSs and a majority of enhancers show cluster-specific changes, we conclude that these regions undergo extensive epigenetic programming during cellular differentiation.

To examine concordance of this cell-type-specific epigenetic regulation with cell-type-specific transcription of lincRNAs, we examined current literature for reports of cell-type-specific transcription of lincRNAs. For a total of 661 GENCODE lincRNAs we could find assertions in the literature about cell-type-specific transcription based on RNA-seq or microarray data. Most publications involved analysis of up to three cell types (see Supplementary Table 4 for details). In contrast to the analysis of relatively large subtrees reported here, previous studies involved differentiated cell types. Nevertheless, 78% of literature assertions assign lincRNAs to the cell types that approximately correspond to one of the subtrees defined here. A total of 52% (266) of those assignments are identical to those reported here. This concordance is significant because only about 1/14 or 7% of the assignments would be expected to coincide by chance (assuming random assignments of lincRNAs to one of the fourteen clusters). We therefore conclude that epigenomic assignments of lincRNAs to specific clusters reported here show significant concordance with transcription-based assignments reported in the literature.

We next asked if the epigenetically programmed regulatory elements associate with lineage-specific mammalian pathways and phenotypes. Towards this goal, we examined mouse phenotypes that result from knockdowns of mouse orthologues of protein-coding genes associated with cluster-specific regulatory elements. Mouse phenotype term enrichment was determined using GREAT tool^24^ (for details see Methods). To ensure that our findings generalize across all three germ layers, we asked the same question for representative lineages descending from mesodermal (T cell), ectodermal (neuronal) and endodermal (gastrointestinal) germ layers. As illustrated in Fig. 2c, the top five mouse phenotype categories were highly lineage-specific for all three representative lineages and for all three classes of regulatory elements—promoters, enhancers and lincRNA TSSs. Similar enrichments can be observed for the majority of other 14 clusters (Supplementary Figs 6 and 7). From this we conclude that cluster-specific histone marks over regulatory regions, including H3K4me1 over lincRNA TSSs have power to accurately identify lineage-specific genes, pathways and phenotypes within all three germ layers.

### Epigenomic footprints of lincRNAs in human ESCs

We next examined epigenomic programming of lincRNA TSSs during differentiation, with a focus on the TSSs that show cluster-specific marks. Towards this goal, for each of the fourteen clusters we identified lincRNA TSSs that show at least one histone mark difference between cluster samples and all other samples, as determined by LIMMA^22^ (see Methods).

**Figure 3:**
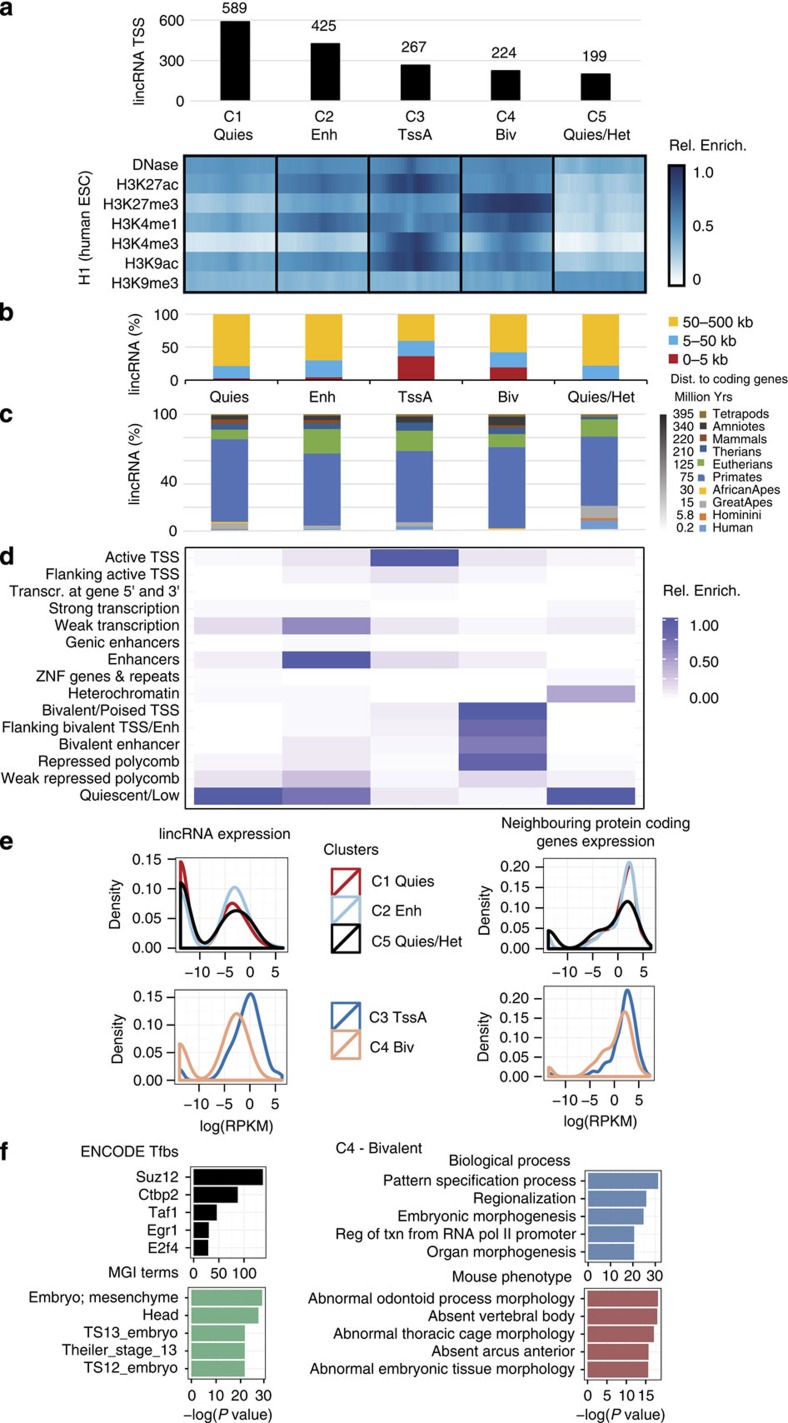
Epigenomic footprints of lincRNA transcription start sites in the H1 embryonic stem cell. lincRNA transcription start sites (lincRNA TSSs) belong to five distinct chromatin state classes. (**a**) LincRNA TSS that had differential histone modification signals (in at least one histone mark) in stem cells were used to perform Spark analysis. Spark performs *k*-means clustering (*k*=5, bin size=100 bps) to group regions that have similar epigenomic footprint. Clustering analysis reveals five distinct classes of lincRNA TSS for H1 stem cells: quiescent (C1: Quies), enhancer (C2: Enh), transcription start site active (C3: TssA), bivalent (C4: Biv) and quiescent/heterochromatin (C5: Quies/Het). Each Spark cluster was subjected to further analyses (**b**–**f**). (**b**) Absolute distance of lincRNA TSS to the nearest protein-coding TSS determined using GREAT basal+extension rule (1 kb downstream+5 kb upstream+up to 500 kb distal). The absolute distances are binned into <5 kb, 5–50 kb and >50–500 kb windows. (**c**) Evolutionary age estimates of lincRNA based on sequence conservation^27^. (**d**) ChromHMM state enrichments of the lincRNA TSS clusters. (**e**) Density function showing expression of lincRNA (left) and neighbouring protein-coding genes in RPKM (reads per kilobase per million) units. (**f**) Enrichment of ENCODE transcription factor binding sites for bivalent lincRNA TSS clusters (hypergeometric tests, *P*<0.0005). Gene ontology terms (blue—biological process, red—mouse phenotype and green—mouse genomic institute (MGI) expression) enrichment of neighbouring protein-coding genes for the bivalent lincRNA TSS cluster. Terms identified using GREAT are significant by both hypergeometric and binomial tests (*P*<0.05).

To establish baseline patterns of chromatin marks in the H1 ESC line, regions 3 Kbp in size centring on the lincRNA TSSs that show ESC-specific marks were clustered by Spark^25^, based on the signal of all histone marks over those regions in H1. As illustrated in Fig. 3a, Spark analysis revealed five clusters corresponding to regions with different combinations of chromatin marks. To further characterize Spark clusters, we examined their enrichment for distinct chromatin states identified by the ChromHMM program^26^. As indicated in Fig. 3d, Spark and ChromHMM independently discovered highly correlated chromatin mark profiles.

The distances between lincRNA TSSs and the closest protein coding gene varied across Spark clusters (Fig. 3b). A majority of lincRNA TSSs belonging to the Quiescent, Active Enhancer and Heterochromatin clusters were located >50 Kbp away from protein-coding genes (Fig. 3b), in contrast to the much more proximal location of those belonging to the active promoter and bivalent clusters.

We then asked whether the lincRNAs with TSSs belonging to different Spark clusters showed differences in their transcription levels. As expected, lincRNAs with TSSs within the Heterochromatin cluster were transcribed at lower levels than other distal lincRNA clusters and those within bivalent cluster were transcribed at a lower level than those within the active promoter cluster (Fig. 3e). These patterns suggest an association between chromatin states and lincRNA transcription.

Using evolutionary dating information for lncRNAs^27^, we asked whether lincRNAs associated with different Spark-defined clusters share similar evolutionary history. As indicated in Fig. 3c, heterochromatin cluster contained the largest fraction of human-specific lincRNAs. The rapid evolution of members of this cluster is consistent with reduced negative selection pressure due to apparent lack of specific function in early embryonic development, as suggested by lower levels of transcription of lncRNAs belonging to the Heterochromatin cluster in the H1 cell line (Fig. 3e).

The bivalent cluster was most stable evolutionarily (Fig. 3c and Supplementary Fig. 8a), consistent with previous findings^27^. As expected, the bivalent cluster showed lower transcription of both lincRNAs and their associated protein-coding genes than the active promoter cluster (Fig. 3e). Enrichment analysis of biological processes and mouse phenotype associated with the bivalent cluster using GREAT tool^24^ revealed strong enrichment for genes involved in developmental regulation (Fig. 3f and Supplementary Fig. 8c). Furthermore, enrichment analysis of ENCODE transcription factor binding^28^ within bivalent cluster C4 in H1 ESC revealed the strongest enrichment for *SUZ12* a key member of the PRC2 (Polycomb repressive complex 2)^29^ and *CTBP2*, a protein known to interact with Polycomb complex members^30^ (Fig. 3f and Supplementary Fig. 8b). These enrichments are consistent with the regulation of the bivalent state by the Polycomb complex.

### Epigenomic regulation of lincRNAs during differentiation

Having established the baseline in ESCs, we next analysed epigenomic programming of lincRNAs upon differentiation. We first examined dynamic epigenomic footprints within the mesodermal germ lineage, using CD8+ T cells as a representative of the lineage. The focus was on a list of variable lincRNA TSSs that showed changes in at least one histone mark along the T-cell subtree. The following three stages of cellular differentiation were analysed: (1) ESC H1; (2) CD34+ haematopoietic stem cell; and (3) fully differentiated CD8+ T cells. By combining chromatin marks at the three stages into a single Spark analysis we aimed to identify groups of lincRNAs TSSs that show similar trajectories of epigenetic programming, each trajectory consisting of a distinct patterns of coordinated changes in histone marks as cells transition between the three stages.

**Figure 4:**
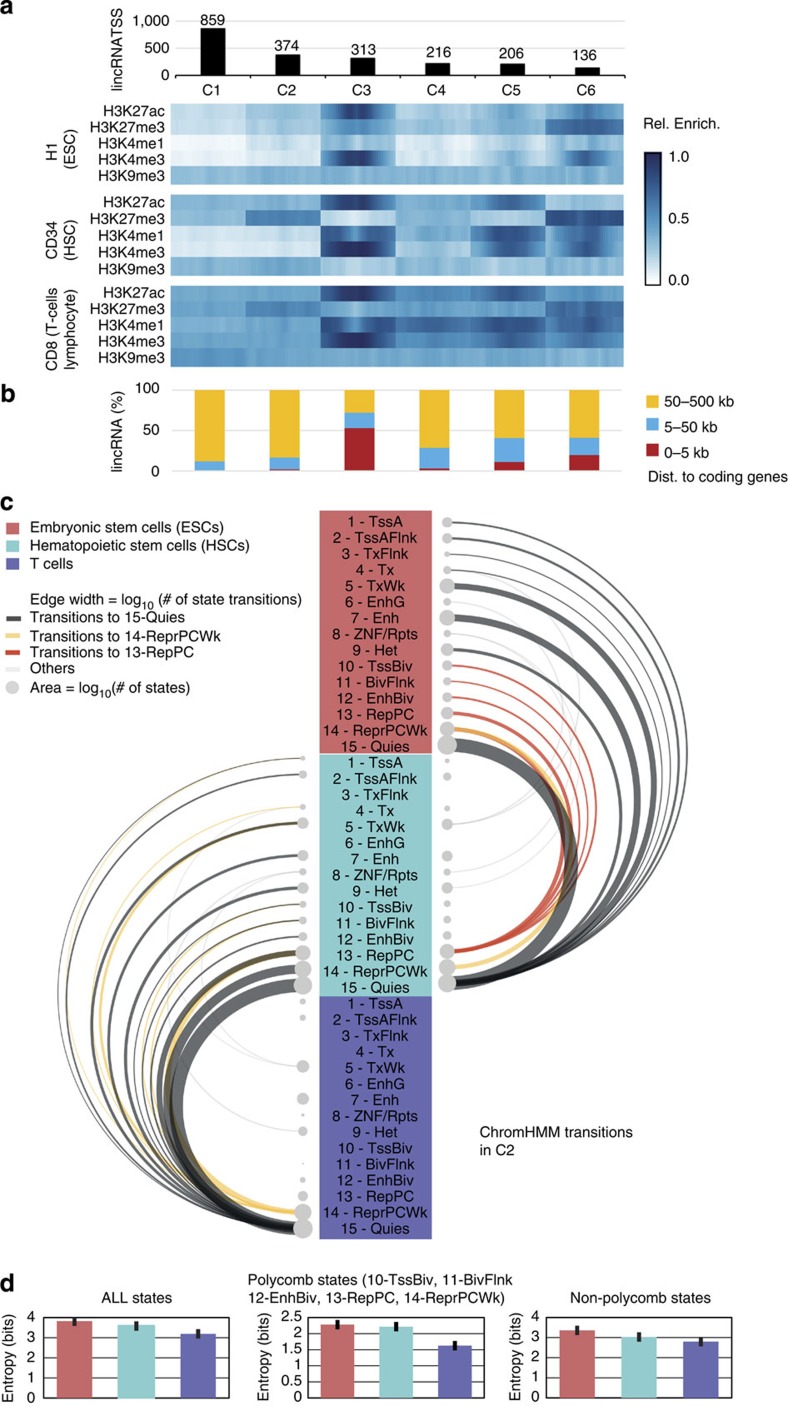
Coordinated changes of chromatin states at lincRNA TSSs during T-cell differentiation. (**a**) Spark clustering reveals coordinated changes in histone marks between human embryonic stem cells (H1), haematopoietic stem cells (CD34+) and T-lymphocyte cells (CD8+). Black bar plot indicates number of lincRNA TSS that show specific pattern of epigenetic programming across the three developmental time points. (**b**) Absolute distance of lincRNA TSS to the nearest protein-coding TSS determined using GREAT basal+extension rule (1 kb downstream+5 kb upstream+upto 500 kb distal). The absolute distances are binned into <5 kb, 5–50 kb and >50–500 kb windows. (**c**) ChromHMM-defined states transition between embryonic stem cells (ESCs) to haematopoietic stem cells (HSCs) and from HSCs to T cells were mapped for lincRNA TSS in C2. Size of the node reflects the number of states and edge width reflects the number of transitions. Transitions >30% relative to each state are shown in the arc diagram. (**d**) Bar plot showing Shannon entropy calculated for all states, Polycomb states or non-Polycomb states for the three developmental time points (ESCs, HSCs and T cells).

As illustrated in Fig. 4a, the largest Spark cluster (C1) consisted of a combination of Quiescent and Heterochromatin states and the second largest (C2) showed signs of Polycomb silencing. The smallest cluster (C6; Fig. 4a) showed bivalent state. In contrast to those generally inactive lincRNA TSSs that were mostly located >50 Kbp away from TSSs of protein-coding genes, the lincRNA TSSs in the active promoter state were generally located within 5 Kbp of TSSs of protein-coding genes (Fig. 4b).

Two clusters (C4 and C5) showed enhancer-like activation patterns on differentiation. Cluster C5 showed an early activation pattern during a transition from ESC state to CD34+ HSCs, while cluster C4 showed activation upon transition from CD34+ HSCs to CD8+ T cells. The results of pathway enrichment analysis using GREAT tool (Supplementary Fig. 9a) are consistent with the different timing of activation: the early activating cluster (C5) is enriched for more generic terms such as ‘immunity’ and ‘leukocyte’ while the late activating cluster (C4) is enriched for more specific terms ‘lymphocyte’ and ‘T cell’. In contrast to both of these activating clusters, the inhibiting clusters C2 and C6 are enriched for pathways leading to other mesodermal lineages, consistent with the well-known Polycomb-mediated inhibition of such pathways during lineage specification^31^.

We next examined changes in transcriptional output for all six clusters for lincRNAs and closest protein-coding genes. In all silencing states (C1-Quescent, C2-Polycomb and C6-Bivalent), decrease in transcription could be observed for both lincRNAs (Supplementary Fig. 9b) and neighbouring protein-coding genes (Supplementary Fig. 9c). The coordinated changes in transcription in lincRNAs and neighbouring protein-coding genes during regional silencing is well established for some Polycomb-regulated loci such as HOX clusters. In contrast, activating clusters C4 and C5 show significant increase in transcription of lincRNAs but no major changes in transcription levels of neighbouring protein-coding genes, suggesting that the lincRNA TSSs marked by H3K4me1, a mark typically associated with active enhancers, may in this case not associate with elevated transcription of closest genes.

### Polycomb regulation of lincRNAs during differentiation

During differentiation along the haematopoietic lineage, a majority of Polycomb-regulated lincRNA TSSs follow a Polycomb-silencing trajectory (cluster C2 in Fig. 4a) while a minority retain bivalent state (cluster C4). Those that are silenced tend to be located further away from protein-coding genes (Fig. 4b).

We next examined the pattern of epigenomic state transitions at lincRNA TSSs along the Polycomb-silencing trajectory (cluster C2 in Fig. 4a and for others see Supplementary Fig. 10). Specifically, at each of the three stages of differentiation (ESCs, HSCs and T cells) we counted lincRNA TSSs that belong to each of the fifteen ChromHMM states and determined counts for each of the 15 × 15 state transitions for both stages of differentiation. Transitions from bivalent (TssBiv, BivFlnk and EnhBiv) to repressed Polycomb (RepPC) state are prominent at the transition from ESCs to HSCs. Strikingly, transitions into repressed Polycomb state practically disappear upon differentiation into T cells, while the transitions into weakly Polycomb repressed (RepPCWk) and particularly into Quiescent (Quies) states gain prominence.

As the bivalent states disappear during differentiation by being resolved into active or inactive states, the combinatorial diversity of histone marks at lincRNA TSSs diminishes and the lincRNA TSSs concentrate within a smaller number of states, particularly within the Quiescent state. This loss of combinatorial diversity may be quantitated using the entropy function over the 15 states. As indicated in Fig. 4d, entropy for lincRNA TSSs in cluster C2 decreases as cells differentiate. Notably, contribution of Polycomb-regulated states is dominant but not exclusive because the diversity of non-Polycomb marks also decreases.

We next examined this trend more broadly and found it across all three germ layers (Supplementary Fig. 11a–c). Specifically, transitions from bivalent (TssBiv, BivFlnk and EnhBiv) to repressed Polycomb (RepPC) state are prominent as the cells differentiate from ESCs towards specific lineages. As in the haematopoietic lineage, transitions into repressed Polycomb state practically disappear during terminal differentiation, while the transitions into weakly Polycomb repressed (RepPCWk) and Quiescent (Quies) states increase.

## Discussion

Expanding previous fragmentary information about tissue-specific role of lincRNAs, we have shown that at least 3,753 (69% examined) lincRNAs show exquisitely tissue-specific epigenomic footprints and strongly associate with cell- and tissue-specific pathways, suggesting developmental or tissue-specific function for this newly discovered class of genes.

Although smaller in absolute number than promoters and enhancers, tissue-specific lincRNAs show slightly higher relative tissue specificity than enhancers and much higher than promoters. The putative functional assignment of the majority of lincRNAs to specific cell and tissue types will inform the targeting of future computational, mechanistic and genetic studies.

The epigenomic footprints also provide mechanistic insights into lincRNA regulation. A larger fraction of lincRNA TSSs is marked by H3K4me1 than H3K4me3 on tissue-specific activation and a significant fraction shows a combined pattern. Increase in the H3K4me1 mark at a lincRNA TSS on differentiation associates with increased transcription of the lincRNA but has little effect on the transcriptional output of proximal protein-coding genes.

Another large fraction of lincRNAs is regulated by the Polycomb complex. The majority of relatively transient ‘bivalent’ states resolve into Polycomb-repressed states during early stages of differentiation, consistent with the established role of PcG proteins in early differentiation^32^. In contrast, as the cells become fully differentiated, transitions into weakly Polycomb-repressed and Quiescent states become dominant. Combinatorial diversity of chromatin states—as measured by entropy—decreases upon cellular differentiation, thus resulting in a smaller number of likely more stable configurations of chromatin marks.

Correlation between transcription levels of lincRNAs and the transcription of proximal protein-coding genes is the strongest in Polycomb-regulated lincRNAs, suggesting regional regulation of chromatin state. The extensive polycomb regulation revealed here is particularly interesting because some lncRNAs are themselves known to regulate polycomb-mediated chromatin silencing^33^ and 20% of them directly associate with Polycomb members^19^. Moreover, 31% of old evolutionarily conserved lncRNAs are regulated by homeoproteins, many of which are known to be in turn regulated by PcG proteins. This suggests potentially complex patterns of feedback loops regulating epigenetic state, local transcription and cellular identify.

In summary, the analyses of dynamic epigenomic footprints suggest an important role for lincRNAs in cellular differentiation. We note that our analyses are an instance of a general methodological template applicable to any class of non-coding RNAs or regulatory elements. To support the broadest application of this methodological template and to enable the scientific community to reproduce and extend analyses reported here we provide a set of integrated on-line tools (see Methods).

We anticipate future studies that will improve on our results in at least three important aspects. First, as the diversity of epigenomically profiled tissues and cells increases, the approach demonstrated in our study may assign even more lincRNAs to specific tissues and cell lineages. Second, significant amount of biological signal in our analyses may have been dampened by the heterogeneity of profiled tissues and by the variation in their cell-type composition. As more pure cell populations become available, we anticipate that an even clearer picture will emerge. Finally, our results strongly suggest cell- and tissue-specific function for thousands of lincRNA genes but do not definitively prove it for any particular gene. We anticipate that the leads provided here will help target future mechanistic and genetic studies required for such definitive proofs.

## Methods

### Chromatin modifications

Data analysis for this paper utilized 111 reference epigenomes within Release 9 of the Human Epigenome Atlas (http://www.epigenomeatlas.org) produced by the Epigenomics Roadmap project^21^. Release 9 consists of 2,804 genome-wide data sets, of which 1,936 are part of the 111 epigenomes analysed here. Informed consents were obtained from all human participants. For the purpose of our analyses, the reference epigenomes were grouped into 99 distinct cell and tissue types (Supplementary Table 5). These reference epigenomes include 1,595 histone modification data sets, 104 DNase data sets, 170 methylation data sets and 67 RNA-Seq data sets, encompassing a total of 111.57 billion mapped sequencing reads corresponding to 5,631-fold coverage of the human genome. All sequenced data sets were uniformly processed by mapping reads onto hg19 assembly of the human genome using Pash 3.0 read mapper^34^. Mapped reads and metadata associated with the sample are archived at GEO describing the origin of the sample, method used for the cell line differentiation, assay, data processing details and quality metrics collected for each experiment. Regulatory elements (enhancers, promoters, lincRNA TSSs and others) with lineage-specific activity patterns, as determined by epigenomic marks, constitute Level 4 data within the Release 9 of the Human Epigenome Atlas and will be distributed through the Atlas portal at the Epigenomics Data Analysis and Coordination Center at Baylor College of Medicine (http://www.epigenomeatlas.org).

### RNA-seq analysis

Analysis of RNA-seq profiles was performed based on the reads mapped by the NIH Epigenomics Roadmap Consortium. Detailed description of RNA-seq library construction and analysis is described elsewhere^35^. JAGuaR^36^ (Junction Alignments to Genome for RNA-seq Reads) pipeline was used to align RNA-seq reads. To increase alignment sensitivity, JAGuaR uses extended reference that includes the genome and reference transcript models that also contains annotated exon–exon junctions. This allows fast and accurate alignment of reads to transcriptome (http://www.bcgsc.ca/platform/bioinfo/software/jaguar). Several QC metrics (intron–exon ratio, intergenic reads fraction, strand specificity, 3′-5′ bias, GC bias and RPKM discovery rate) were calculated to assess quality of RNA-seq library. RPKM for all genes were calculated using total number of reads aligned onto all merged exons normalized by total exonic length. Epigenome Atlas Release 9 will include files containing RPKM values for all annotated exons for both coding and non-coding genes based on Gencode v10 annotations^37^.

### Chromatin state learning

A 15-state ChromHMM model was inferred by the Consortium^38^ across all the 127 (111 reference epigenomes plus 16 ENCODE epigenomes) containing five core chromatin marks (H3K4me1, H3K4me3, H3K36me3, H3K9me3 and H3K27me3). The model was used to annotate the epigenomes and was used for chromatin state enrichment and transition analyses.

### Genomic regions of interest

The functional annotations used were as follows: (1) lincRNA genes, exons, introns, 3′UTR, miRNA, TSS, 3 kb windows around lincRNA TSSs were based on the GENCODE v10 annotations; (2) CpG islands were obtained from the UCSC table browser; and (3) ChromHMM enhancer-like regions based on 15 chromatin state models.

### Epigenomic data slices

Epigenomic slicer tool within the Epigenomic Toolset was used to obtain data slices for specific mark-ROI combinations. A data slice for a set of samples is a set of arrays, one array per sample, each array consisting of average signals of a specific histone mark over a given set of regions of interest (ROIs). Data slices were calculated for across all 99 distinct cell types profiled in Roadmap Epigenome Atlas, for 40 pairwise combinations of five different core marks (H3K4me1, H3K4me3, H3K36me3, H3K27me3 and H3K9me3) and eight different ROIs—lincRNA gene body, lincRNA TSS (+/−3 kb around TSS), miRNA, protein-coding gene body, protein-coding exons, protein-coding 3′UTR, protein-coding promoter (+/−3 kb around TSS) and ChromHMM-predicted^26^ enhancers. The ChromHMM enhancer predictions were based on 15 chromatin state models learned across 127 epigenomes (19 ENCODE epigenomes plus 111 epigenomes within Release 9 of the Human Epigenome Atlas). Data slices were output in a matrix format (samples as columns and rows as regions) suitable for importing into R statistical programming language for downstream analysis.

### Hierarchical clustering

Data matrices generated using Genboree Workbench Epigenomic slicer tool was used for Hierarchical clustering of epigenomes. Data matrices were quantile normalized between epigenomes. PVclust package^39^ in R was used to perform hierarchical clustering. We used Pearson’s correlation as distance measure and average as the clustering method. Tree generated using the package was formatted using newick tree annotation format. Circular representation of the hierarchical clustering was generated using GraphLan^40^ phylogenetic tree viewer.

### Confirmation score for trees and mark-ROI combinations

Confirmation scores were calculated for each of the 40 different mark-ROI combinations (Marks: H3K4me1, H3K4me3, H3K9me3, H3K27me3 and H3K36me3; ROIs: lincRNA, lincRNA TSSs, chromHMM enhancers, 3′UTR, promoter, miRNA, exon and gene body). Within the Release 9 of the Epigenome Atlas, a total of 99 cell types and tissues were represented by at least one sample with all five histone mark profiles. However, several of the histone modification assays have a different number of technical or biological replicates. Therefore, to compare clusters across assay types, we performed sub-sampling of replicates. Specifically, we randomly selected one replicate from each sample containing multiple replicates, effectively generating 99-sample data slices for each of 40 independent mark-ROI combinations (Fig. 1a). To ensure optimal and unbiased replicate selection, we repeated the sub-sampling process 50 times for each mark-ROI combination. Thus, instead of generating a single 99-sample data slice for each mark-ROI combination, we generated 50 data slices for each of the 40 mark-ROI combinations, a total of 2,000 data slices for the set of 99 samples. We constructed 2,000 trees, one for each of the 2,000 data slices by pvclust, using correlation as the distance measure and the average method for hierarchical clustering. Trees were output in Newick file format. For each of the 2,000 Newick files, the frequency of each subtree in the 2,000 trees was determined (a subtree that is present in 1,800 out of the 2,000 trees would get a frequency score of 1,800/2,000=0.9). Each of the 2,000 Newick files was then scored by taking the average frequency score for all of its subtrees. Because each mark-ROI combination was represented by 50 trees, score for each mark-ROI was calculated by taking the average of the 50 trees. The scores, rounded to a single decimal are in Fig. 1a.

### Determination of differentially modified histone regions

For each of the 14 highly recurrent subtrees in Fig. 1b and for each of the 40 mark-ROI combinations, we identified regions where the samples within the subtree differ from others. We determined these regions by comparing epigenomes within the subtree against all epigenomes outside of it. To find differentially modified histone regions we used LIMMA^22^ (Linear Model for Microarray Analysis) tool. As input we provided epigenomic data slices (see Method Section 2.1), specifying the epigenomes that are subjected for comparison, and setting significance threshold of FDR-corrected *P* value<0.05. LIMMA identifies regions with significant signal differences between epigenomes within the subtree versus epigenomes outside of it.

### Enrichment analyses

Gene ontology enrichment analysis was performed using GREAT^24^. Cell- and tissue-specific regions were assigned to nearby protein-coding genes based on GREAT’s basal plus extension rule for regulatory regions (proximal: 5 kb upstream, 1 kb downstream, plus distal up to 500 kb). Annotated terms selected from enrichment analysis were significant by both hypergeometric and binomial tests (*P*<0.05). ENCODE TFBS^28^ (transcription factor binding site) enrichment analysis was performed using an in-house script. Enrichment of transcription factor binding sites over a set of ROIs was performed using a hypergeometric distribution model. Such model assumed as background the binding sites of all transcription factors probed by ENCODE, and tested whether binding sites of a particular transcription factors had a stronger tendency of occurring in the set of ROIs than binding sites of all other transcription factors combined. A TFBS and a particular ROI were said to co-occur when they overlapped by at least 1 bp.

### Clustering ROIs based on multiple epigenomic marks

Spark^25^, multi-signal comparative analysis tool, was used to cluster ROIs based on multiple epigenomic marks. Spark performs K-means clustering of loci based on multiple marks within a single cell type (as in Fig. 3) or multiple marks over multiple developmental stages (as in Fig. 4). ROIs that correspond to individual clusters were downloaded in the form of annotation tracks for the purpose of downstream analyses such as pathway enrichment analyses by GREAT. GREAT’s basal plus extension rule was used to associate cis-elements (such as TSSs of lincRNAs) with protein-coding gene for gene-set enrichment analysis.

### Chromatin state transitions

To study epigenomic trajectories of lincRNA during differentiation, we restricted chromatin state transitions to lincRNA TSS (3 kb window). We used a 15-state model to study state transitions across three developmental time points—ESCs, intermediate and fully differentiated cell type. For each developmental transition, from stem cells to intermediate and from intermediate to fully differentiated cell type, we calculated the number of all pairs of chromatin state transitions (15 states in ESCs × 15 states in intermediate=225 pairs of state transitions) and performed row normalization to the each resulting 15 × 15 matrix. Arcdiargram package in R was used to render the graph of state transitions across three developmental time points. The graph nodes represent states in stem cells, intermediate and differentiated cell type. The edge widths indicate proportion of transitions normalized based on each state. An entropy score for distribution across the 15 states was calculated for each of the three developmental time points.

### Integrated tool set to reproduce and extend reported analyses

The analyses reported in this paper can be reproduced using the data and tools described above. For reader’s convenience, the tools were also integrated into the Epigenomic Toolset within the Genboree Workbench. A number of instructive tool set use cases are accessible at http://genboree.org/theCommons/projects/aminv-natcomm-2015/wiki. Use cases specifically describe the types of analyses reported here and provide step-by-step instructions for performing analyses using Clustering, LIMMA, Spark and Enrichment tools.

## Additional information

**Accession codes:** All data generated by the NIH Roadmap Epigenomics Project have been deposited in the NCBI Gene Expression Omnibus under project accession codes- GSE16256, GSE16368, GSE17312, GSE18927, GSE19465, GSE25246, GSE25247, GSE25248, GSE25249. GEO accession numbers for all the experiments can be accessed at the NIH Roadmap Epigenomics Project Data listings http://www.ncbi.nlm.nih.gov/geo/roadmap/epigenomics/ or through the Atlas portal at the Epigenomics Data Analysis and Coordination Center at Baylor College of Medicine http://www.genboree.org/epigenomeatlas/index.rhtml.

**How to cite this article:** Amin, V. *et al*. Epigenomic footprints across 111 reference epigenomes reveal tissue-specific epigenetic regulation of lincRNAs. *Nat. Commun.* 6:6370 doi: 10.1038/ncomms7370 (2015).

## Supplementary Material

Supplementary Information Supplementary Figures 1-11, Supplementary Tables 1-5, Supplementary Methods and Supplementary References

Supplementary Dataset 1 Genomic coordinates of transcription start sites for lincRNA with the H3K4me1 chromatin mark.

Supplementary Dataset 2 Genomic coordinates of enhancers with the H3K4me1 chromatin mark

Supplementary Dataset 3 Genomic coordinates of transcription start sites for lincRNA with the H3K4me3 chromatin mark

Supplementary Dataset 4 Genomic coordinates of promoters with the H3K4me3 chromatin mark
