# Supplementary material for: Epigenomic footprints across 111 reference epigenomes reveal tissue-specific epigenetic regulation of lincRNAs
Source: Nat Commun. 2015 Feb 18;6:6370. doi: 10.1038/ncomms7370 (PMC4335353; doi:10.1038/ncomms7370)
Supplement: Supplementary Information — Supplementary Figures 1-11, Supplementary Tables 1-5, Supplementary Methods and Supplementary References [file ncomms7370-s1.pdf]

Supplementary Figure 1

a) 40 combinations of histone marks and regions of interests (mark-ROI)

Example of confirmation score calculation of one (H3K4me3-Protein coding TSS) out of 40 mark-ROI combination shown below

Histone Mark	Region of Interest (ROI)
**H3K4me3**	**Protein coding gene TSS (+/- 1500 bp)**
H3K4me1	Protein coding Exons
H3K27me3	Protein coding 3’ UTR
H3K9me3	Protein coding gene body
H3K36me3	ChromHMM defined Enhancers
	lincRNA
	lincRNA TSS (+/- 1500 bp)
	miRNA

b) Data Slice over single mark (H3K4me3)-ROI (protein coding promoters) combination

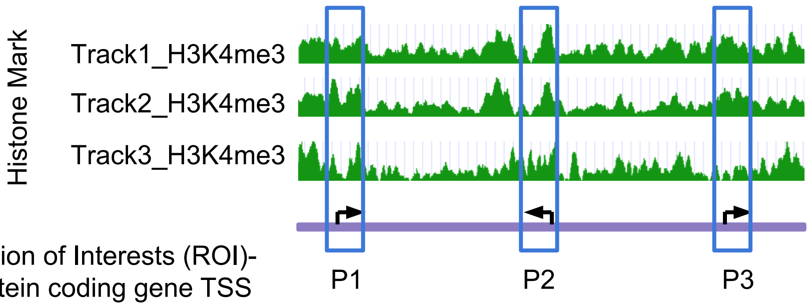

c) Calculate average signal or reads for each track and region

	Track 1	Track 2	Track 3
P 1	0.8	0.7	0.3
P 2	0.65	0.6	0.7
P 3	0.8	0.8	0.2

d) Hierarchical clustering to group samples based on similarity

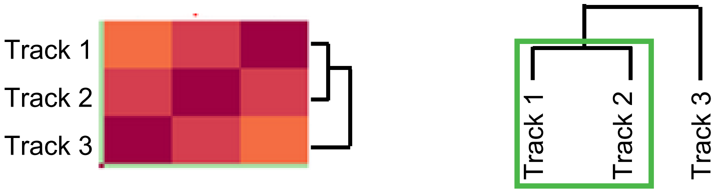

e) To calcuate the confirmation score, subtrees are first determined for a given tree. In this example, there are two, Track1-Track2 and Track1-Track2-Track3 grouped togather. Frequency of these subtrees occuring in trees generated from a different mark-ROI combinations are determined. The confirmation score is calcuated by taking sum of the subtrees frequencies.

Supplementary Figure 1. Clustering and confirmation score calculation. **(a)** Clustering was performed using 40 different possible combinations of marks and regions of interests (mark-ROI combinations). Calculations for one mark-ROI combination are illustrated in the following example with three samples (**b-d**). **(b)** Epigenomic data slice tool within Genboree Workbench was used to obtain an average mark signal in a set of samples (represented by tracks) for a specific set of regions of interests (ROIs). **(c)** Epigenomic data slice tool outputs a data matrix of average signals per ROI per track (sample). **(d)** Hierarchical clustering of epigenomes was performed using a correlation-based distance measure and the average method for hierarchical clustering. A total of 2000 trees were constructed, 50 replicate trees for each of the 40 different mark-ROI combination. A replicate tree consisted of one randomly selected technical or biological replicate for each of the 99 sample types. Each subtree (such as the track1-track2 cluster illustrated in this figure) was assigned a score equal to the fraction of the 2000 trees that contain the subtree. Conformation score for a mark-ROI combination is an average for all the 50 trees obtained for this mark-ROI combination.

Supplementary Figure 2

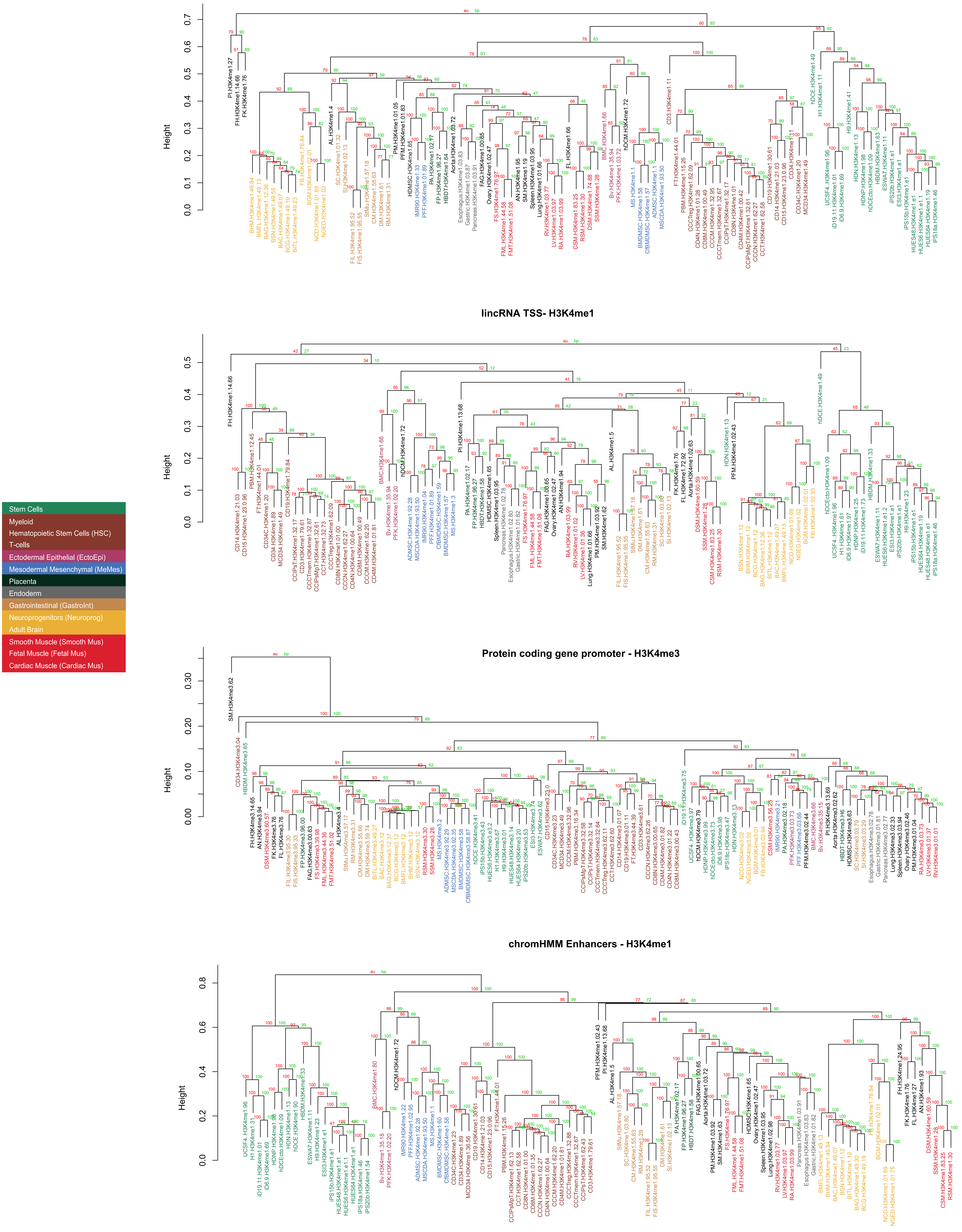

Supplementary Figure 2. Measure of support for all the branch points in the trees with different histone mark-ROI combinations. The leaves of the tree are colored according to the samples assigned to a cell type cluster. Trees were constructed for the following 4 mark-ROI combinations: (**a**) average H3K4me1 signal over 3'UTR (**b**) average H3K4me1 histone modification signal over 3000bp windows centered on lincRNA transcription start sites, (**c**) average H3K4me3 signal over 3000bp windows centered on transcription start sites of protein coding genes, and (**d**) average H3K4me1 signal over ChromHMM defined enhancers.

Supplementary Figure 3

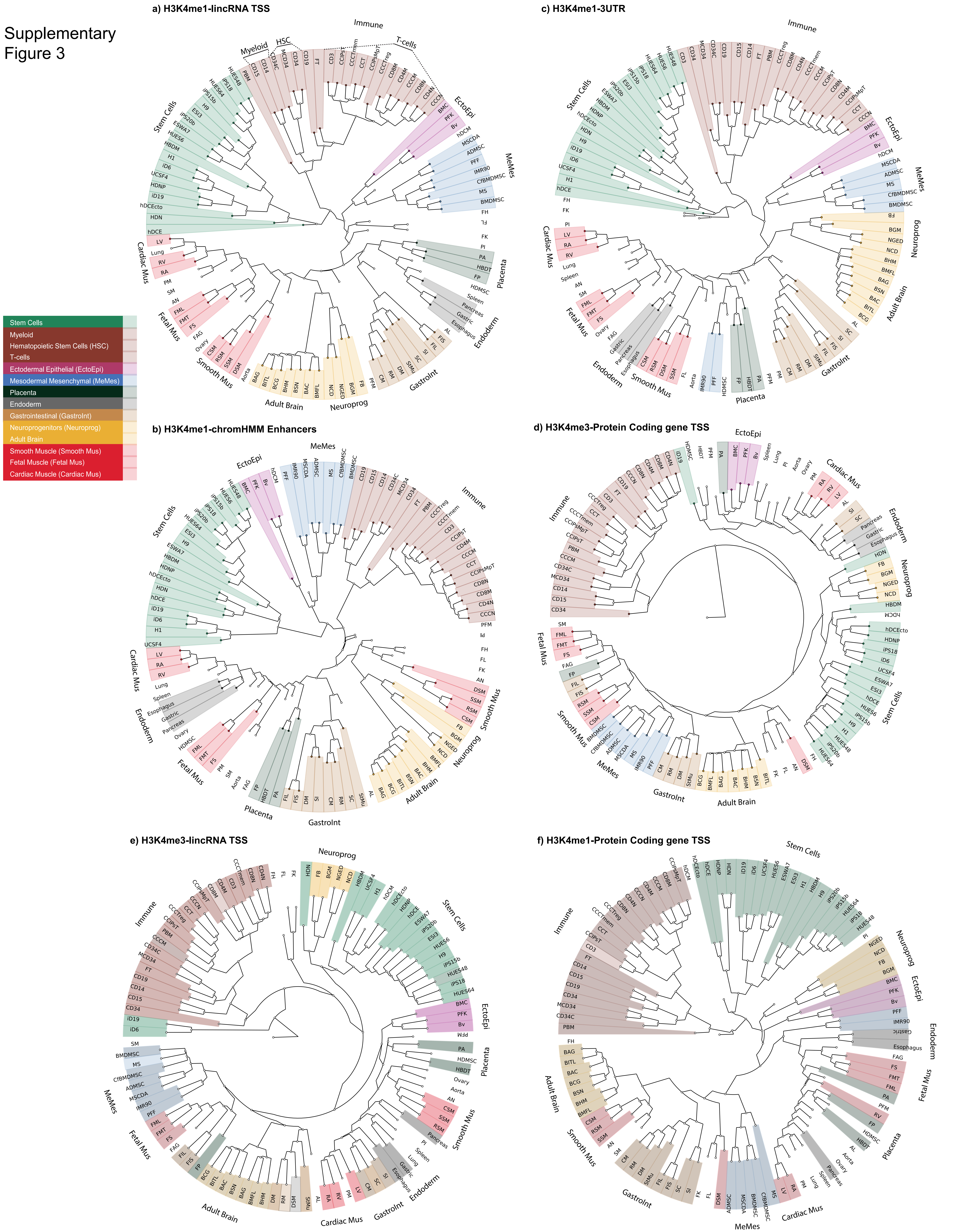

Supplementary Figure 3. Circular dendrogram displays of the trees constructed using four different histone mark-ROI combinations. For all four trees, the leaves are colored according to fourteen clusters that are highly recurrent and also correspond to established cell and tissue types. Description of the assay track and cell type abbreviation are described in Supplementary Table 5 and 6. Trees were constructed using following mark-ROI combinations: (**a**) average H3K4me1 histone modification signal over 3000bp windows centered on lincRNA transcription start sites, (**b**) average H3K4me1 signal over ChromHMM[1,2] defined enhancers, (**c**) average H3K4me1 signal over 3'UTR, and (**d**) average H3K4me3 signal over 3000bp windows centered on transcription start sites of protein coding genes, (**e**) average H3K4me3 signal over 3000bp windows centered on lincRNA transcription start sites, and (**f**) average H3K4me1 signal over 3000bp windows centered on transcription start sites of protein coding genes.

Supplementary Figure 4

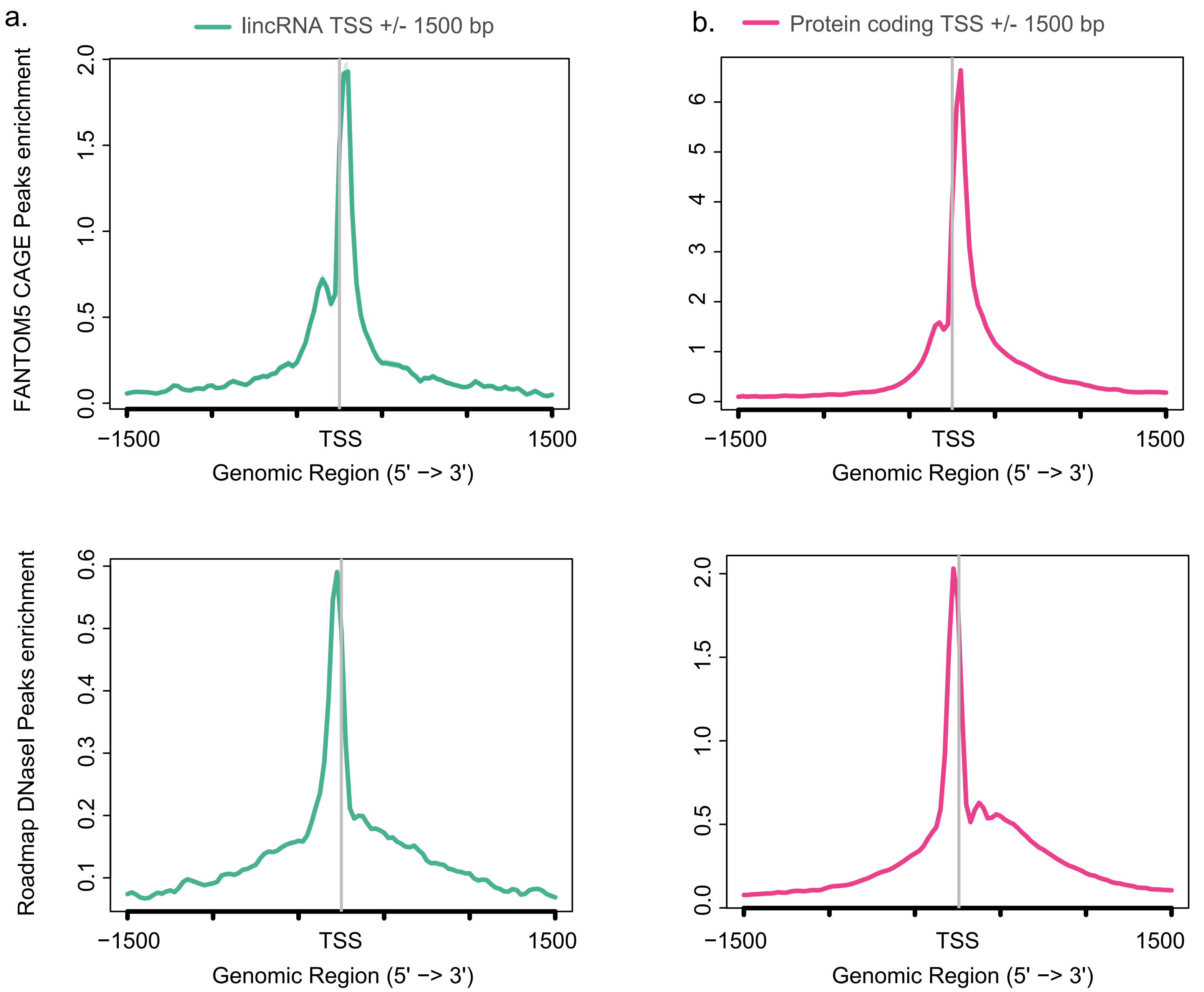

Supplementary Figure 4. Enrichment of FANTOM5 CAGE tags generated across multiple cell types[2] and Roadmap Epigenome Project generated DNase hypersensitivity reads generated across multiple cell types at, (**a**) GENCODE defined lincRNA transcription start sites (+/- 1500 bp), and (**b**) GENCODE defined protein coding genes transcription start sites (+/- 1500 bp).

Supplementary Figure 5

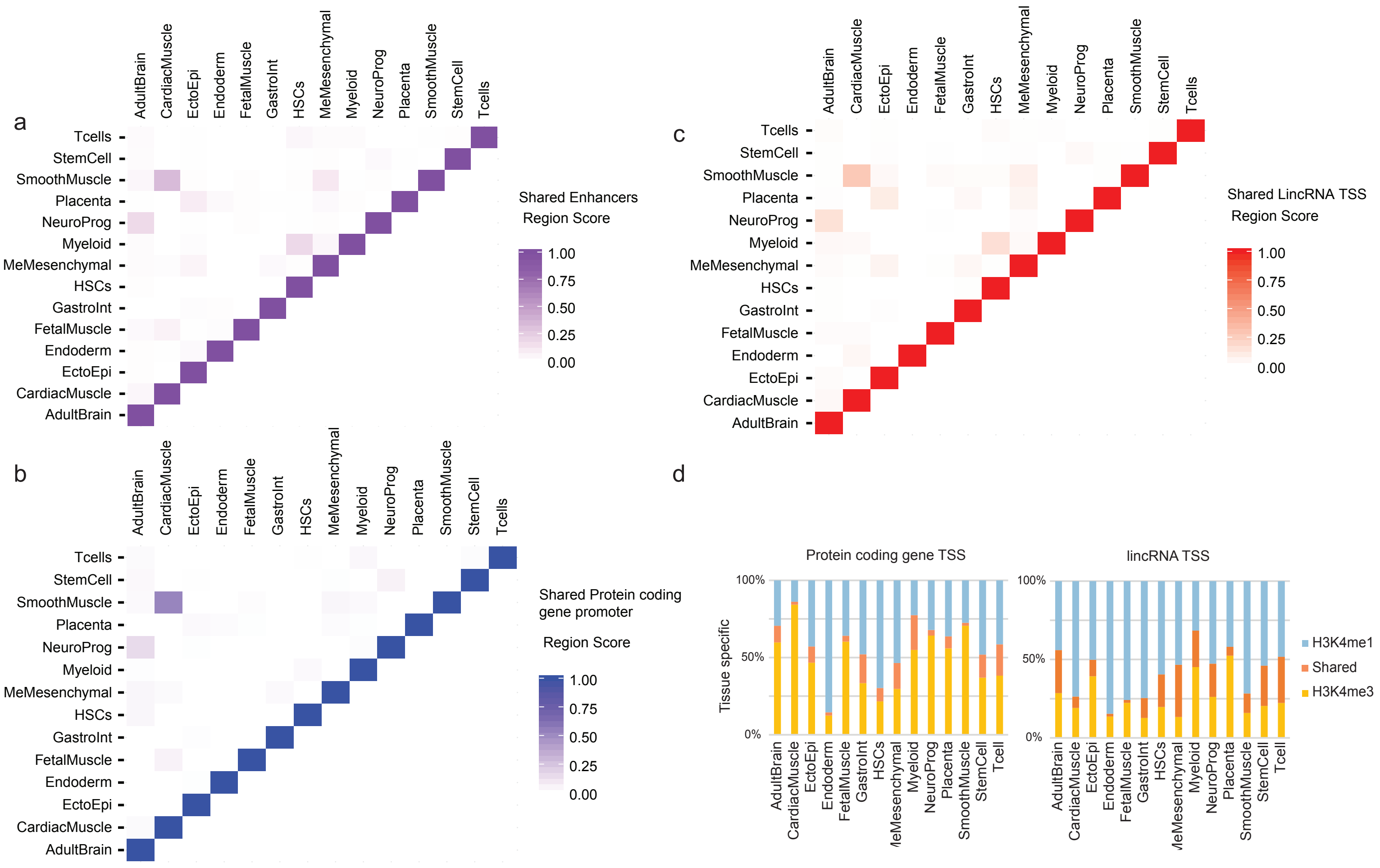

Supplementary Figure 5. (**a-c**) Enrichment of cluster-specific regulatory regions within and across clusters: (**a**) ChromHMM defined enhancers, (**b**) Protein coding gene promoters (+/- 3kb TSS), and (**c**) lincRNA TSS (+/- 3kb TSS). Note higher sharing of regions by developmentally related clusters. (**d**) Proportion of tissue specific lincRNA TSS and protein coding gene TSS having either H3K4me1 or H3K4me3 or having both marks.

Supplementary Figure 6

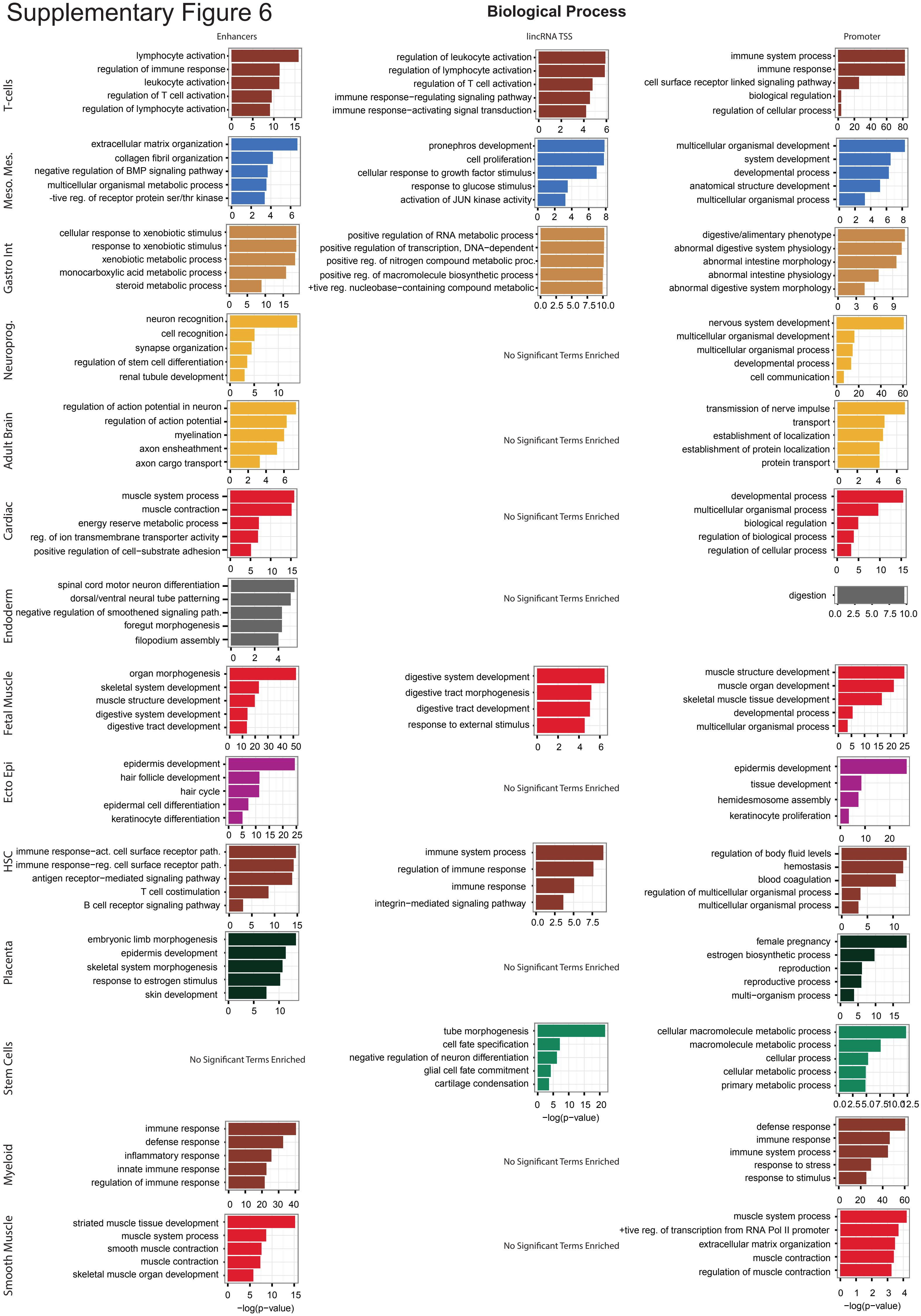

Supplementary Figure 6. Enrichment of top five **biological processes** for cluster-specific regulatory regions - (**a**) Enhancers based on differential H3K4me1 mark, (**b**) lincRNA TSS based on differential H3K4me1 mark, and (**c**) protein coding genes promoters based on differential H3K4me3 mark.

Supplementary Figure 7

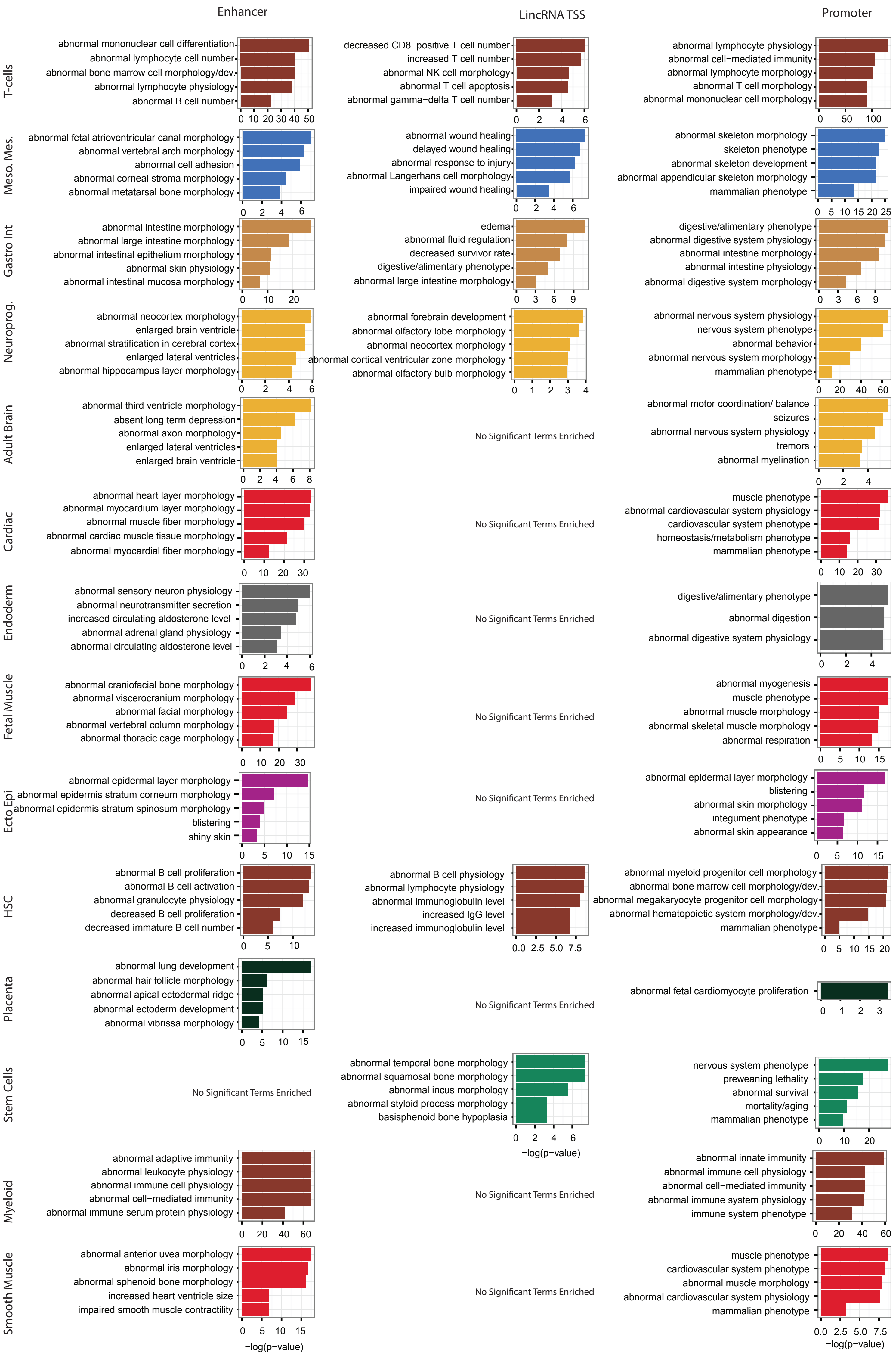

Supplementary Figure 7. Enrichment of top five **mouse phenotype** for cluster-specific regulatory regions - (**a**) Enhancers based on differential H3K4me1 mark, (**b**) lincRNA TSS based on differential H3K4me1 mark, and (**c**) protein coding genes promoters based on differential H3K4me3 mark.

Supplementary Figure 8

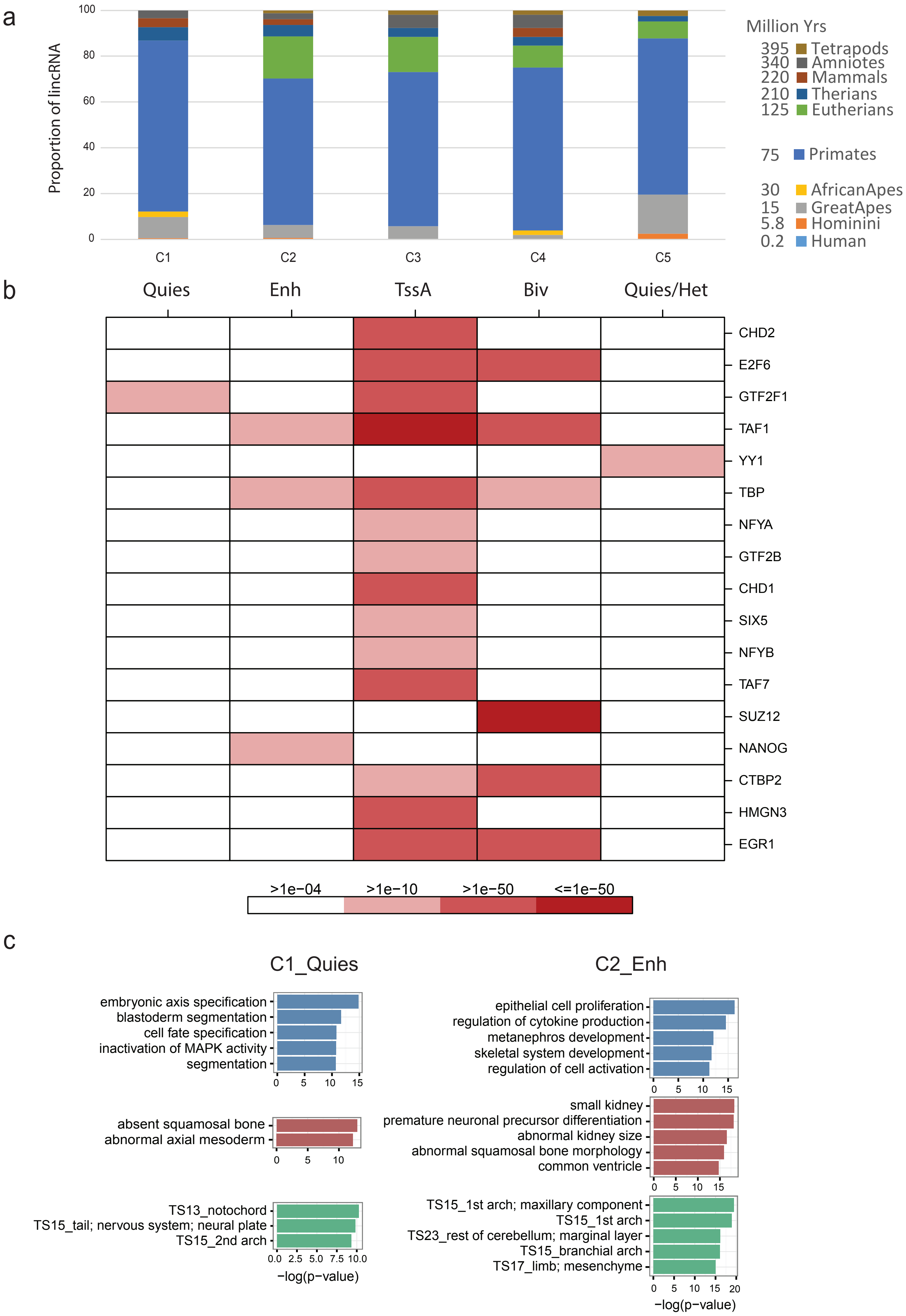

Supplementary Figure 8. (**a**) Evolutionary expression conservation of lincRNA[3]. (**b**) Enrichment of ENCODE transcription factor binding sites for all lincRNA TSS clusters (hypergeometric tests, $p < 0.0005$). (**c**) Gene ontology terms (blue - biological process, red - mouse phenotype, and green - mouse genomic institute (MGI) expression) enrichment of neighboring protein coding genes for C1 - Quiescent and C2 - Enhancer. No terms were enriched for C3 - Active TSS and C5 - Quies/Hetrochromatin. Terms identified using GREAT are significant by both hypergeometric and binomial tests ($p < 0.05$).

Supplementary Figure 9

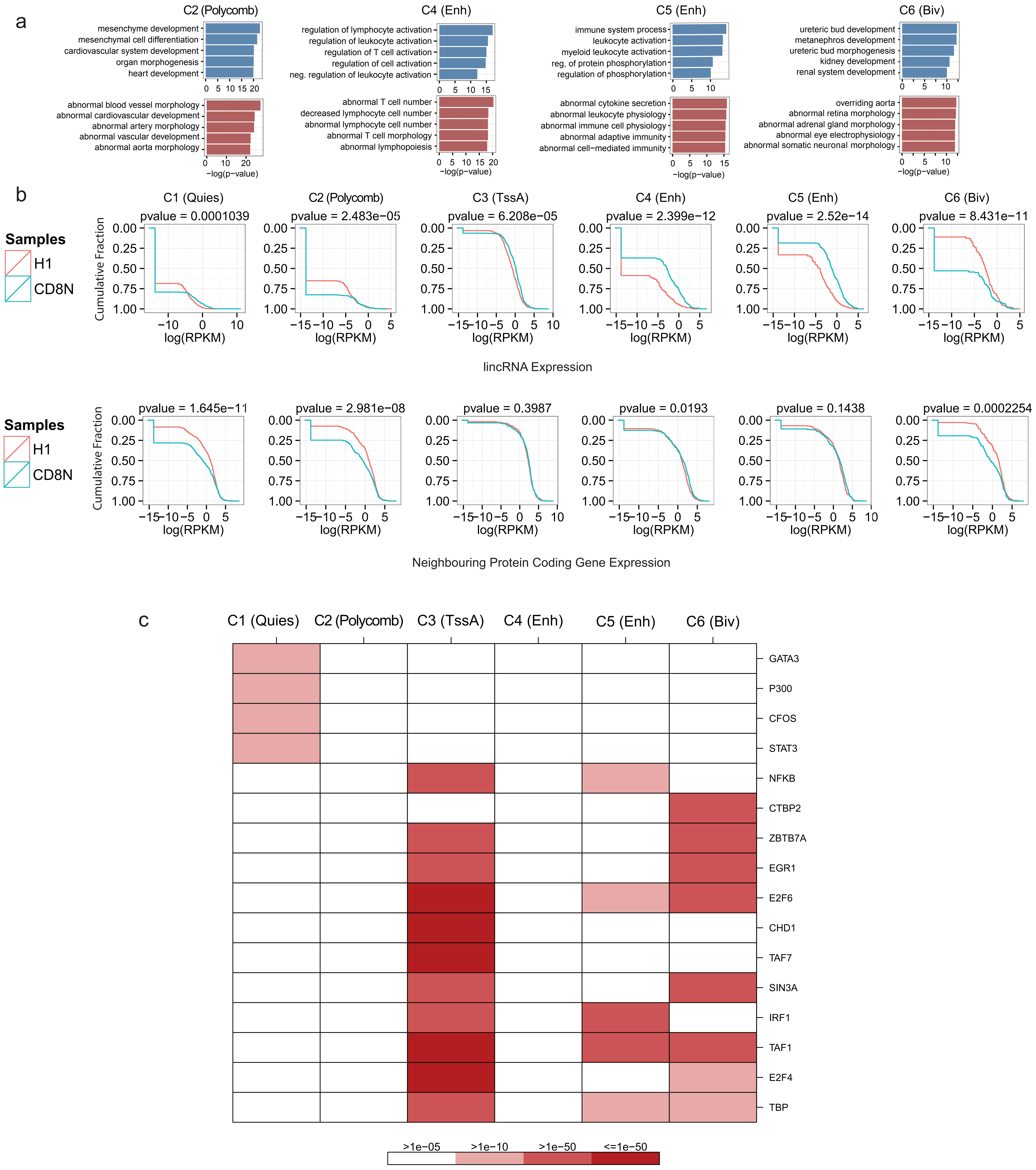

Supplementary Figure 9. (**a**) Ontology term enrichment (blue - GO biological process and red - mouse phenotype) of neighboring protein coding genes for active enhancer (C4 and C5), polycomb regulated (C2), and bivalent (C6) lincRNA TSS clusters in Figure 4. Terms were identified using GREAT and are significant by both hypergeometric and binomial tests ($p < 0.05$). (**b**) Changes in transcription levels of lincRNAs and nearby protein coding genes as cells transition from H1 to CD8. Cumulative density plots of RPKM values for lincRNAs within specific the 6 clusters in Figure 4 and for associated protein coding genes (GREAT basal + extension rule). P-values are calculated using Kolmogorov-Smirnov test and are shown on top of each cumulative density plots. (**c**) Enrichment of ENCODE transcription factor binding sites for all lincRNA TSS clusters in Figure 4.

Supplementary Figure 10

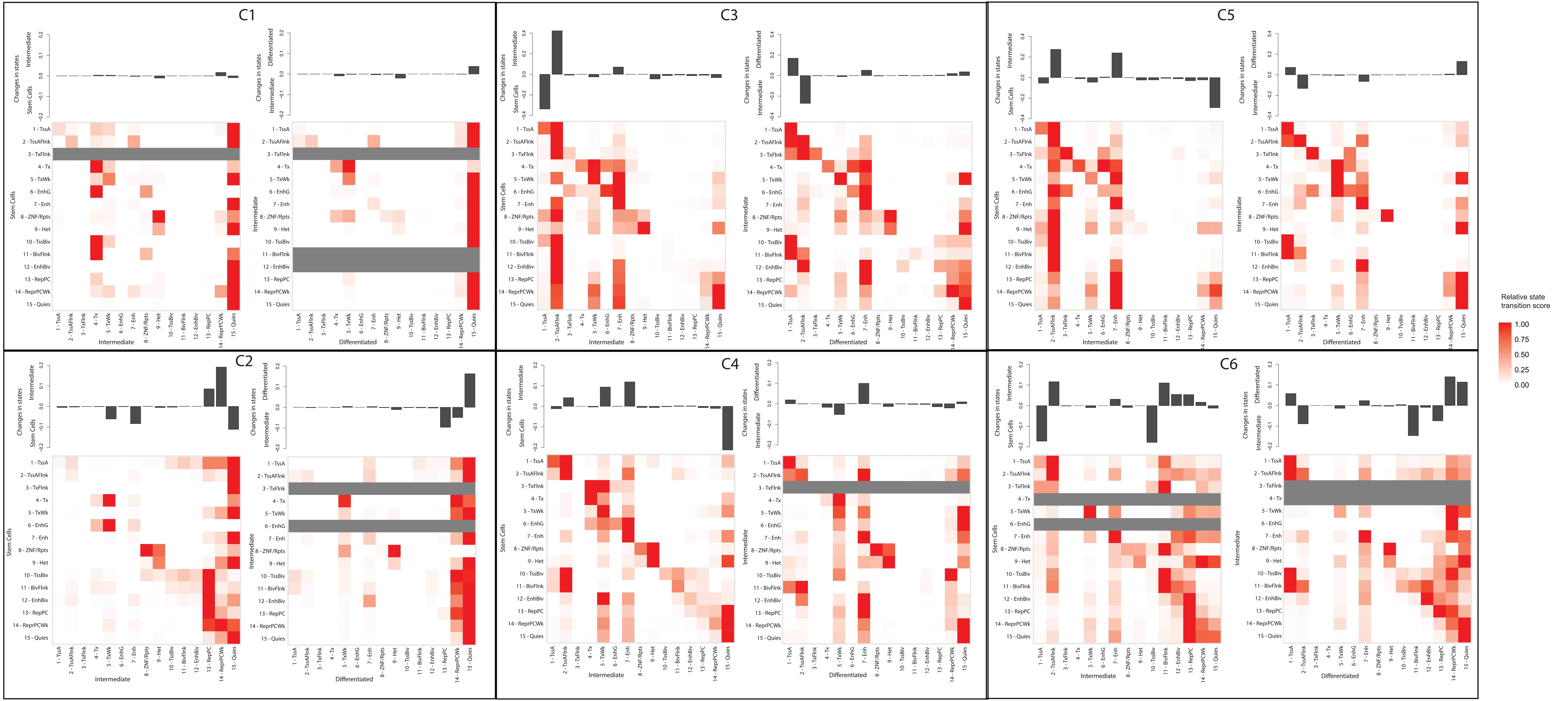

Supplementary Figure 10. Chromatin state transitions across all lincRNA TSS clusters in Figure 4 during T-cell differentiation. For all the clusters labeled C1-C6 in Figure 4, heatmap (row normalized) shows the chromHMM defined state transitions frequency for each state from embryonic stem cells (stem cells) to hematopoietic stem cells (intermediate) and second heatmap shows chromatin state transitions frequency from hematopoietic stem cells (intermediate) to T-cells (Differentiated). Above each heatmap, barplot shows cumulative changes of a state during transition.

Supplementary Figure 11

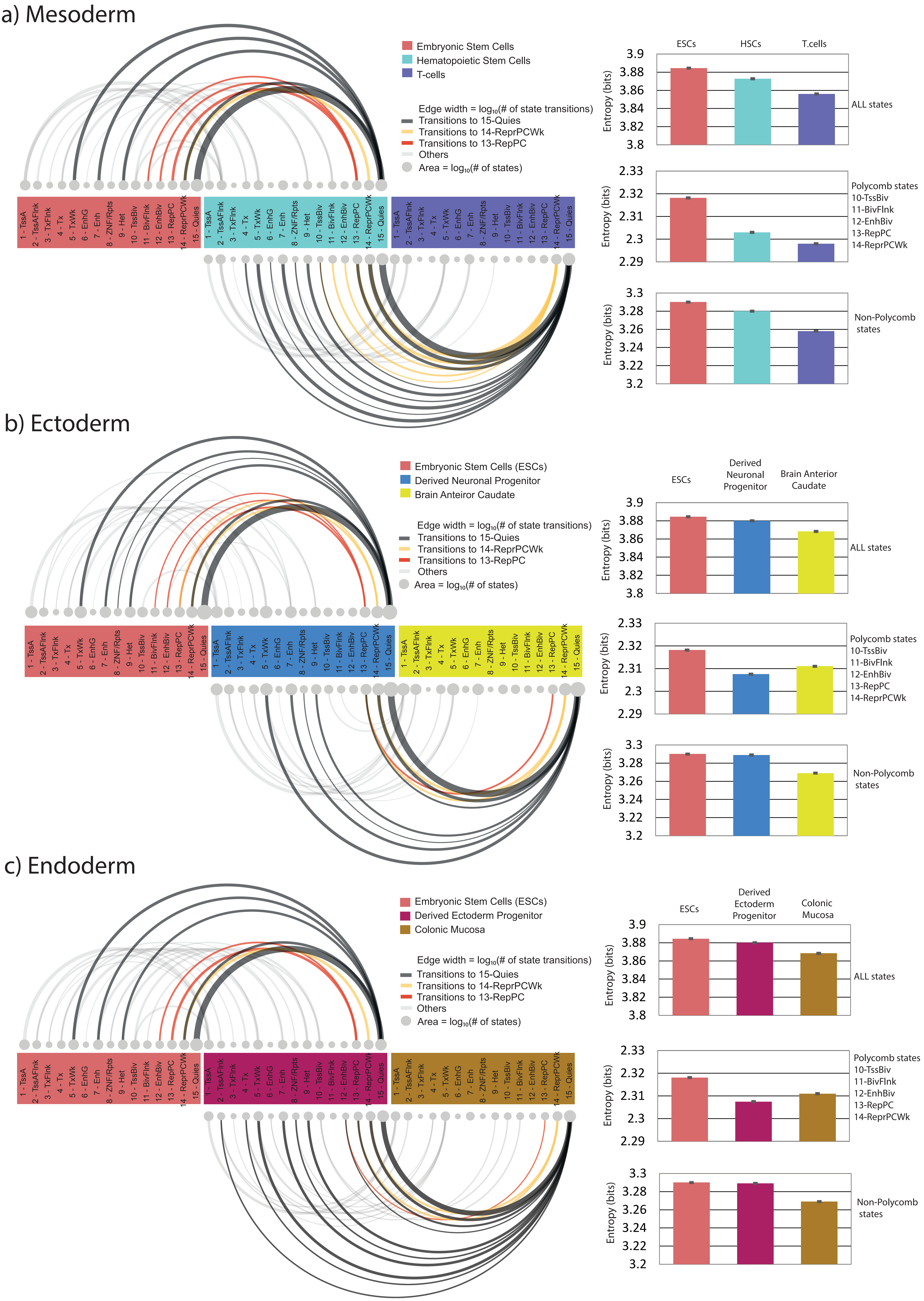

Supplementary Figure 11. Chromatin state transitions at lincRNA TSS in cell lineages representing all three germ layers: (**a**) T-cells (Mesoderm); (**b**) Neuronal (Ectoderm); and (**c**) Colonic mucosa (Endoderm). For representatives of each germ layer, chromatin state transitions were mapped between embryonic stem cells to intermediate state and from intermediate state to terminally differentiated cell type. Size of the nodes in the arc diagram reflects the number of lincRNA TSSs in particular states and edge widths reflects the counts of transitions. Only transitions with counts greater than 30% of each state are shown in the arc diagram. For each germ layer and each developmental stage, Shannon entropy was calculated, as shown in barplots on the right. Entropy was calculated separately for all states, Polycomb states, and non-Polycomb states.

Supplementary Table 1 - Confirmation score summary of frequently occurring subtrees from trees with different chromatin mark-region of interest combination. Bootstrap score of the subtrees for top chromatin mark-region of interest combination trees are also reported in the table.

		Bootstrap score			
**Clusters**	**Confirmation Score**	**3UTR**	**lincRNATSS**	**Promoter**	**Enhancer**
Adult Brain	100	100	100	100	100
Immune	81.2	100	100	-	100
Fetal Muscle	80.8	97	99	100	100
Smooth Muscle	65.6	100	100	-	100
Neuroprogenitor	64	100	98	100	100
Gastrointestinal	54.8	100	93	-	100
Mesodermal Mesenchymal	53.6	-	100	-	99
Ectodermal Epithelial	43.6	92	100	-	100
Placenta	37.6	100	100	-	100
Endoderm	20.4	92	99	83	100
Stem Cell	12	92	-	-	100
Cardiac Muscle	6.8	100	-	99	100

Supplementary Table 2 - Counts of cell/tissue cluster specific regulatory regions.

Cell /Tissue Clusters	H3K4me3-lincRNA TSS	H3K4me1 -lincRNA TSS	H3K4me1-H3K4me3-lincRNA TSS	H3K4me1-Enhancer	H3K4me3-Promoter	H3K4me1-Promoter	H3K4me1-H3K4me3 Promoter
1	1929 (35.2%)	2819 (51.5%)	2937 (53.6%)	294105 (58.8%)	7426 (37.1%)	8629 (43%)	10095 (50.5%)
2	126 (2.3%)	329 (6%)	762 (13.9%)	45882 (9.2%)	703 (3.5%)	568 (2.8%)	2973 (14.9%)
3	2 (0%)	17 (0.3%)	54 (1%)	1714 (0.3%)	19 (0.1%)	4 (0.02%)	243 (1.2%)
4	0 (0%)	0 (0%)	4 (0.1%)	9 (0%)	0 (0%)	0 (0%)	3 (0.01%)
>4	3427 (62.5%)	2319 (42.3%)	1727 (31.5%)	158290 (31.7%)	11859 (59.3%)	11378 (56.9%)	6693 (33.5%)

Supplementary Table 3 - Summary statistic of chromHMM enhancer mark signal in lineage specific genes in comparison to non-lineage specific genes.

		Adult Brain	Cardiac	Ectodermal Epithelial	Endoderm	Fetal Muscle	Gastrointestinal	Hematopoietic Stem Cells	Mesodermal Mesenchymal	Myeloid	Neuroprogenitor	Placenta	Smooth Muscle	Stem Cells	T cells
Number of cluster specific H3K4me1 marks in protein coding gene bodies	H3K4me3 specific protein coding gene promoters	7722	2284	610	227	59	857	459	3052	1483	2298	178	381	13805	16731
	Non-H3K4me3 specific protein coding gene promoters (Avg. of 1000 simulations)	2086	313	127	56	8	126	84	710	257	231	27	38	5015	7251
	**Fold Change**	**3.7**	**7.3**	**4.8**	**4.1**	**7.4**	**6.8**	**5.5**	**4.3**	**5.8**	**9.9**	**6.6**	**10.0**	**2.8**	**2.3**
Number of cluster specific H3K4me1 marks in lincRNA gene bodies	H3K4me1 specific lincRNA TSS	545	126	256	54	89	151	230	1131	173	329	76	240	2535	1871
	Non-H3K4me1 specific lincRNA TSS (average of 1000 simulations)	80	16	39	10	4	19	37	255	18	48	6	22	904	324
	**Fold Change**	**6.8**	**7.9**	**6.6**	**5.4**	**22.3**	**7.9**	**6.2**	**4.4**	**9.6**	**6.9**	**12.7**	**10.9**	**2.8**	**5.8**

Supplementary Table 4 - Summary of the concordance of cell/tissue cluster specific lincRNAs based on epigenetic regulation from this manuscript to current reports of cell type specific transcription of lincRNAs.

Cell type	Organism	Analysis details	Numbers overlapping to GENCODE lincRNA identifiers	Matched to corresponding lineages	Other lineages	Common across multiple lineages	Ref
T-cell	Mouse	Out of 1524 lincRNAs associated with T-cell functions, 400 regions mapped to human coordinates hg19 using liftOver tool (min_match = 0.65). These 400 regions mapped to 47 lincRNAs from GENCODE lincRNA annotations.	47	21	13	13	4
Stem Cell	Mouse	Out of 137 lincRNAs that altered mESC regulatory proteins, 41 regions mapped to human coordinates hg19 using liftOver tool (min_match = 0.65). These 41 mapped regions overlapped to 4 lincRNAs from GENCODE lincRNA annotations.	4	2	1	1	5
Various	Mouse	Out of 18 lincRNA regions, 7 mapped to human coordinates hg19 using liftOver tool (min_match = 0.65). These 7 mapped regions overlapped to 2 lincRNA from GENCODE lincRNA anntoations. Two lincRNAs are linc-Pint and Mdgt. linc-Pint KO was found to be specific for several developmental lineages (Adult Brain, HSCs, Mesodermal Mesenchymal, and Tcells), another lincRNA Mdgt is specific to stem cells. linc-Pint knockout resulted in runnted mice and Mdgt knockout mice had lethal phenotypes.	2	2	0	0	6
Fibroblasts, immortalized lymphoblastoid cell lines, and primary T cells	Human	Umbilical cord and blood cell cord sample were collected from 204 newborn and used to derive three cell lines - primary fibroblasts, primary T cells, and lymphoblastoid cell lines. The study determined 63 (fibroblasts), 67 (lymphoblastoid cell lines), and 60 (Tcells) GENCODE lincRNAs expressed in cell specific manner.	190	78	59	53	7

Various	Human	From this study, 6 cell-types do represent to one of 14 cell/tissue clusters (Placenta, adipose+breast, colon, heart, white blood cell, and brain) while the others that did not are (liver, kidney, skeletal muscle, adrenal+lymphNode, prostate, lung, thyroid, and testes).	210	83	84	43	[8]
Colon	Human	This study found CCAT1-L, lncRNA 515kb upstream of MYC expresses specifically in colorectal cancers and regulates MYC expression through long-range chromatin looping	1	1	0	0	[9]
Stem cell	Human	This study showed lincRNA-RoR regulates Oct4, Nanog, and Sox2 in human embrynoic stem cell self-renewal	1	1	0	0	[10]
Stem cell	Human	Based on RNA-Seq datasets from ENCODE, systematically identified 300 human embryonic stem cell lincRNAs. Of these 206 overlapped with GENCODE lincRNAs.	206	78	90	38	[11]
		**Total**	661	266	247	148	

Supplementary Table 5 - Description of Cell Type Abbreviations.

Abbreviation	Sample Type	Cluster	GSM
ADMSC	Adipose Derived Mesenchymal Stem Cell Cultured Cells	Mesodermal Mesenchymal	GSM772748
AN	Adipose Nuclei	-	GSM669975
AL	Adult Liver	-	GSM621654
Aorta	Aorta	-	GSM1059448
BMDMSC	Bone Marrow Derived Mesenchymal Stem Cell Cultured Cells	Mesodermal Mesenchymal	GSM621402
BAG	Brain Angular Gyrus	Adult Brain	GSM772770
BAC	Brain Anterior Caudate	Adult Brain	GSM772830
BCG	Brain Cingulate Gyrus	Adult Brain	GSM670033
BGM	Brain Germinal Matrix	Neuralprogenitor	GSM806939
BHM	Brain Hippocampus Middle	Adult Brain	GSM669962
BITL	Brain Inferior Temporal Lobe	Adult Brain	GSM670036
BMFL	Brain Mid Frontal Lobe	Adult Brain	GSM773014
BSN	Brain Substantia Nigra	Adult Brain	GSM669941
BMC	Breast Myoepithelial Cells	Ectodermal Epithelial	GSM613870
Bv	Breast vHMEC	Ectodermal Epithelial	GSM613868
CD14	CD14 Primary Cells	Immune (Myeloid)	GSM1102793
CD15	CD15 Primary Cells	Immune (Myeloid)	GSM773038
CD19	CD19 Primary Cells	Immune	GSM1027296
CD3	CD3 Primary Cells	Immune (T-cells)	GSM1058778
CD34C	CD34 Cultured Cells	Immune (HSC)	GSM486707
CD34	CD34 Primary Cells	Immune (HSC)	GSM486707
CD4M	CD4 Memory Primary Cells	Immune (T-cells)	GSM772924
CD4N	CD4 Naive Primary Cells	Immune (T-cells)	GSM772869
CCCN	CD4+ CD25- CD45RA+ Naive Primary Cells	Immune (T-cells)	GSM772851
CCCM	CD4+ CD25- CD45RO+ Memory Primary Cells	Immune (T-cells)	GSM772928
CCIPsMpT	CD4+ CD25- IL17- PMA-Ionomycin stimulated MACS	Immune (T-cells)	GSM997268

	purified Th Primary Cells		
CCIPsT	CD4+ CD25- IL17+ PMA-Ionomcyin stimulated Th17 Primary Cells	Immune (T-cells)	GSM997224
CCT	CD4+ CD25- Th Primary Cells	Immune (T-cells)	GSM997243
CCCTreg	CD4+ CD25+ CD127- Treg Primary Cells	Immune (T-cells)	GSM772973
CCCTmem	CD4+ CD25int CD127+ Tmem Primary Cells	Immune (T-cells)	CCCTmem H3K4me1 32 67
CD8M	CD8 Memory Primary Cells	Immune (T-cells)	GSM772873
CD8N	CD8 Naive Primary Cells	Immune (T-cells)	GSM773032
CfBMDMSC	Chondrocytes from Bone Marrow Derived Mesenchymal Stem Cell Cultured Cells	Mesodermal Mesenchymal	GSM670000
CSM	Colon Smooth Muscle	Smooth Muscle	GSM772972
CM	Colonic Mucosa	Gastrointestinal	GSM916046
DM	Duodenum Mucosa	Gastrointestinal	GSM621403
DSM	Duodenum Smooth Muscle	Smooth Muscle	GSM772837
ESI3	ES-I3 Cell Line	Stem Cells	GSM772789
Esophagus	Esophagus	Endoderm	GSM1120349
ESWA7	ES-WA7 Cell Line	Stem Cells	GSM537641
FAG	Fetal Adrenal Gland	-	GSM1102796
FB	Fetal Brain	Neuralprogenitor	GSM806934
FH	Fetal Heart	-	GSM772732
FIL	Fetal Intestine Large	Gastrointestinal	GSM1058775
FIS	Fetal Intestine Small	Gastrointestinal	GSM1058776
FK	Fetal Kidney	-	GSM621409
FL	Fetal Lung	-	GSM706853
FML	Fetal Muscle Leg	Fetal Muscle	GSM1058777
FMT	Fetal Muscle Trunk	Fetal Muscle	GSM1160197
FP	Fetal Placenta	Placenta	GSM1102795
FS	Fetal Stomach	Fetal Muscle	GSM1102794
FT	Fetal Thymus	Immune	GSM1027298
Gastric	Gastric	Endoderm	GSM1013147
HBDT	H1 BMP4 Derived Trophoblast Cultured Cells	Placenta	GSM752977
H1	H1 Cell Line	Stem Cells	GSM537679

HDMSC	H1 Derived Mesenchymal Stem Cells	-	GSM767347
HDNP	H1 Derived Neuronal Progenitor Cultured Cells	Stem Cells	GSM1013146
H9	H9 Cell Line	Stem Cells	GSM667626
HDN	H9 Derived Neuron Cultured Cells	Stem Cells	HDN H3K4me1 13
hDCE	hESC Derived CD184+ Endoderm Cultured Cells	Stem Cells	GSM772971
hDCEcto	hESC Derived CD56+ Ectoderm Cultured Cells	Stem Cells	GSM997254
hDCM	hESC Derived CD56+ Mesoderm Cultured Cells	Stem Cells	GSM916070
HUES48	HUES48 Cell Line	Stem Cells	GSM669954
HUES6	HUES6 Cell Line	Stem Cells	GSM669885
HUES64	HUES64 Cell Line	Stem Cells	GSM772800
IMR90	IMR90 Cell Line	Mesodermal Mesenchymal	GSM521897
iD19.11	iPS DF 19.11 Cell Line	Stem Cells	GSM752979
iD6.9	iPS DF 6.9 Cell Line	Stem Cells	GSM706073
iPS15b	iPS-15b Cell Line	Stem Cells	GSM772767
iPS18a	iPS-18a Cell Line	Stem Cells	GSM773028
iPS20b	iPS-20b Cell Line	Stem Cells	GSM772804
LV	Left Ventricle	Cardiac Muscle	GSM906404
Lung	Lung	-	GSM1059443
MSCDA	Mesenchymal Stem Cell Derived Adipocyte Cultured Cells	Mesodermal Mesenchymal	GSM772745
MCD34	Mobilized CD34 Primary Cells	Immune (HSC)	GSM621451
MS	Muscle Satellite Cultured Cells	Mesodermal Mesenchymal	GSM621759
NCD	Neurosphere Cultured Cells Cortex Derived	Neuralprogenitor	GSM817230
NGED	Neurosphere Cultured Cells Ganglionic Eminence Derived	Neuralprogenitor	GSM707008
Ovary	Ovary	-	GSM1013148
Pancreas	Pancreas	Endoderm	GSM1013149
PI	Pancreatic Islets	-	GSM1127087
PFF	Penis Foreskin Fibroblast Primary Cells	Mesodermal Mesenchymal	GSM817234
PFK	Penis Foreskin Keratinocyte Primary Cells	Ectodermal Epithelial	GSM941736
PFM	Penis Foreskin Melanocyte Primary Cells	-	GSM941730
PBM	Peripheral Blood Mononuclear Primary Cells	Immune	GSM613884
PA	Placenta Amnion	Placenta	GSM1127129
PM	Psoas Muscle	-	GSM956021

RM	Rectal Mucosa	Gastrointestinal	GSM621659
RSM	Rectal Smooth Muscle	Smooth Muscle	GSM915335
RA	Right Atrium	Cardiac Muscle	GSM1059445
RV	Right Ventricle	Cardiac Muscle	GSM1059445
SC	Sigmoid Colon	Gastrointestinal	GSM956023
SM	Skeletal Muscle	-	GSM621686
SI	Small Intestine	Gastrointestinal	GSM1120350
Spleen	Spleen	-	GSM910577
StMu	Stomach Mucosa	Gastrointestinal	GSM772907
SSM	Stomach Smooth Muscle	Smooth Muscle	GSM621642
Thymus	Thymus	Immune	GSM1059446
UCSF4*	UCSF-4* Cell Line	Stem Cells	GSM1127080

Supplementary Methods

Detailed information about data processing and replicates

Two types of processing methods were applied and highly concordant results (in terms of clusters and biological pathway enrichments identified) were reported in this and--in a highly abbreviated form--in the Consortium paper[2]. Both processing methods were applied to the same data set (the same subset of Release 9 of the Human Epigenome Atlas described in Supp. Table 6). The first method (described in detail in the Consortium paper and available at http://www.broadinstitute.org/~anshul/projects/roadmap/signal/36bpReadLensFiltered/pval/) did not group samples (biological replicates) across different donors, resulting in 111 samples. For the purpose of ChromHMM analysis (identical ChromHMM analysis results used in this and the Consortium paper), read counts obtained by the first method were computed in non-overlapping 200 bp bins across the entire genome. Each bin was discretized into two levels, 1 indicating enrichment and 0 indicating no enrichment. The binarization was performed by comparing ChIP-seq read counts to corresponding whole-cell extract control ("input") read counts within each bin and using a Poisson p-value threshold of 1e-4. The second processing method grouped biological replicates (samples from the same tissue type from multiple donors) into 99 distinct samples and applied quantile normalization prior to clustering (Figure 1) and Bayesian linear modeling implemented in the Limma algorithm for the purpose of identifying biological pathway enrichments (Figure 2). Clusters reported in Figure 1 and tissue-specific pathways such as those reported in Figure 2 were corroborated using ChromHMM analysis and the first processing method.

The vast majority (81) of 99 cell types had multiple technical or biological replicates for at least one of the five chromatin marks and 59 of them had replicates for all five marks. All nine groups of clusters in Figure 1 could be reproduced without single-replicate samples. At the finer level of resolution, all fourteen clusters could be reproduced after eliminating single-replicate samples with the exception of myeloid and fetal muscle clusters because they consisted exclusively of single-replicate samples. The two clusters group correctly at the higher level--myeloid cluster belongs to the immune cluster group that also includes T-cell and hematopoietic stem cell clusters; fetal muscle cluster belongs to the muscle cluster group that also includes heart and smooth muscle-- suggesting that the clustering was unlikely to have been affected by sequencing issues.

Supplementary References

1. Ernst, J. *et al.* Mapping and analysis of chromatin state dynamics in nine human cell types. *Nature* **473,** 43–49 (2011).
2. Roadmap Epigenomics Consortium *et al.* Integrative Analysis of 111 reference human epigenomes. *Companion Manuscript* (2015).
3. Necsulea, A. *et al.* The evolution of lncRNA repertoires and expression patterns in tetrapods. *Nature* **505,** 635–640 (2014).
4. Hu, G. *et al.* Expression and regulation of intergenic long noncoding RNAs during T cell development and differentiation. *Nat. Immunol.* **14,** 1190–1198 (2013).
5. Guttman, M. *et al.* lincRNAs act in the circuitry controlling pluripotency and differentiation. *Nature* **477,** 295–300 (2011).
6. Sauvageau, M. *et al.* Multiple knockout mouse models reveal lincRNAs are required for life and brain development. *eLife* **2,** (2013).
7. Popadin, K., Gutierrez-Arcelus, M., Dermitzakis, E. T. & Antonarakis, S. E. Genetic and Epigenetic Regulation of Human lincRNA Gene Expression. *Am. J. Hum. Genet.* **93,** 1015–1026 (2013).
8. Cabili, M. N. *et al.* Integrative annotation of human large intergenic noncoding RNAs reveals global properties and specific subclasses. *Genes Dev.* **25,** 1915–1927 (2011).
9. Xiang, J.-F. *et al.* Human colorectal cancer-specific CCAT1-L lncRNA regulates long-range chromatin interactions at the MYC locus. *Cell Res.* **24,** 513–531 (2014).
10. Wang, Y. *et al.* Endogenous miRNA sponge lincRNA-RoR regulates Oct4, Nanog, and Sox2 in human embryonic stem cell self-renewal. *Dev. Cell* **25,** 69–80 (2013).
11. Tang, X. *et al.* Systematically profiling and annotating long intergenic non-coding RNAs in human embryonic stem cell. *BMC Genomics* **14 Suppl 5,** S3 (2013).
